# Pluripotency retention and exogenous mRNA introduction in planarian stem cells in culture

**DOI:** 10.1016/j.isci.2023.106001

**Published:** 2023-01-20

**Authors:** Kai Lei, Wenya Zhang, Jiajia Chen, Sean A. McKinney, Eric J. Ross, Heng-Chi Lee, Alejandro Sánchez Alvarado

**Affiliations:** 1 Stowers Institute for Medical Research, Kansas City, MO 64110, USA; 2 Westlake Laboratory of Life Sciences and Biomedicine, Key Laboratory of Growth Regulation and Translational Research of Zhejiang Province, School of Life Sciences, Westlake University, Hangzhou, Zhejiang 310024, China; 3 Institute of Biology, Westlake Institute for Advanced Study, Hangzhou, Zhejiang 310024, China; 4 Howard Hughes Medical Institute, Stowers Institute for Medical Research, Kansas City, MO 64110, USA; 5 Department of Molecular Genetics and Cell Biology, University of Chicago, Chicago, IL 60637, USA

**Keywords:** Biological sciences, Molecular biology, Stem cells research

## Abstract

Planarians possess naturally occurring pluripotent adult somatic stem cells (neoblasts) required for homeostasis and whole-body regeneration. However, no reliable neoblast culture methods are currently available, hindering mechanistic studies of pluripotency and the development of transgenic tools. We report robust methods for neoblast culture and delivery of exogenous mRNAs. We identify optimal culture media for the short-term maintenance of neoblasts *in vitro* and show via transplantation that cultured stem cells retain pluripotency for two days. We developed a procedure that significantly improves neoblast yield and purity by modifying standard flow cytometry methods. These methods enable the introduction and expression of exogenous mRNAs in neoblasts, overcoming a key hurdle impeding the application of transgenics in planarians. The advances in cell culture reported here create new opportunities for mechanistic studies of planarian adult stem cell pluripotency, and provide a systematic framework to develop cell culture techniques in other emerging research organisms.

## Highlights

• Planarian neoblast pluripotency can be maintained in optimized culture conditions
• SiRNeoblasts serve as an alternative source of transplantable neoblasts
• Cultured SiRNeoblasts provide a system to examine transgene tools for planarians
• Exogenous mRNA can be expressed in differentiating SiRNeoblasts *in vitro*

## Graphical abstract

**Figure:**
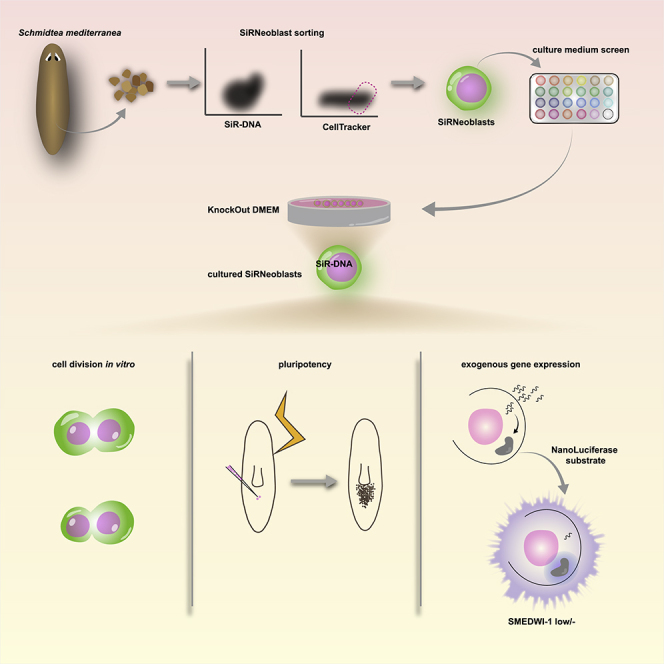

## Introduction

While the control of pluripotency in animals has been examined in the germline and embryonic and induced pluripotent stem cells, no naturally occurring adult pluripotent stem cells have yet been identified in the roundworm, fly, fish, or rodent model systems.^1^^,^^2^^,^^3^^,^^4^ By contrast, planarian flatworms, and acoels uniquely harbor an adult stem cell population, collectively termed neoblasts,^5^ which includes a pluripotent subpopulation of clonogenic neoblasts^6^^,^^7^^,^^8^ that enable whole-body regeneration and apparently limitless capacity for tissue homeostasis. The freshwater species *Schmidtea mediterranea* is a model for planarian development, regeneration, and, in particular, pluripotency in long-lived adult animals.^9^ While regulators of pluripotency have been identified in neoblasts and studied using the RNA interference,^10^^,^^11^^,^^12^ a lack of reliable culture methods and transgenesis limit *in vivo* exploration of adult stem cell pluripotency.^13^ Indeed, published commentary has called for establishing reliable, standardized neoblast culture methods as an essential step in developing transgenic studies of planarians.

While genetic transformation strategies typically exploit early-stage embryos or cultured stem cells,^14^^,^^15^^,^^16^^,^^17^^,^^18^^,^^19^^,^^20^ planarians instead reproduce asexually through neoblast proliferation and differentiation.^21^ These cells can be transplanted into hosts lacking neoblasts (e.g., after lethal irradiation) to repopulate stem cells and rescue the host within one month of the irradiation.^6^^,^^8^ Thus, transforming DNA or RNA into neoblasts before transplantation could produce transgenic planarians and subsequently enable significant breakthroughs in understanding the control of pluripotency in animals. However, despite numerous efforts, no reports currently describe the thriving culture of neoblasts or genetic modification of these animals. Based on the tremendous potential for planarian neoblasts to fill significant gaps in our understanding of regeneration in higher animals, we aimed to establish a robust culture method for pluripotent neoblasts that also enables efficient screening for successful delivery and transgenic expression of exogenous DNA or RNA.

## Results

### KnockOut Dulbecco's Modified Eagle Medium with 5% CO_2_ can maintain the pluripotency of neoblasts *in vitro*

**Figure 1:**
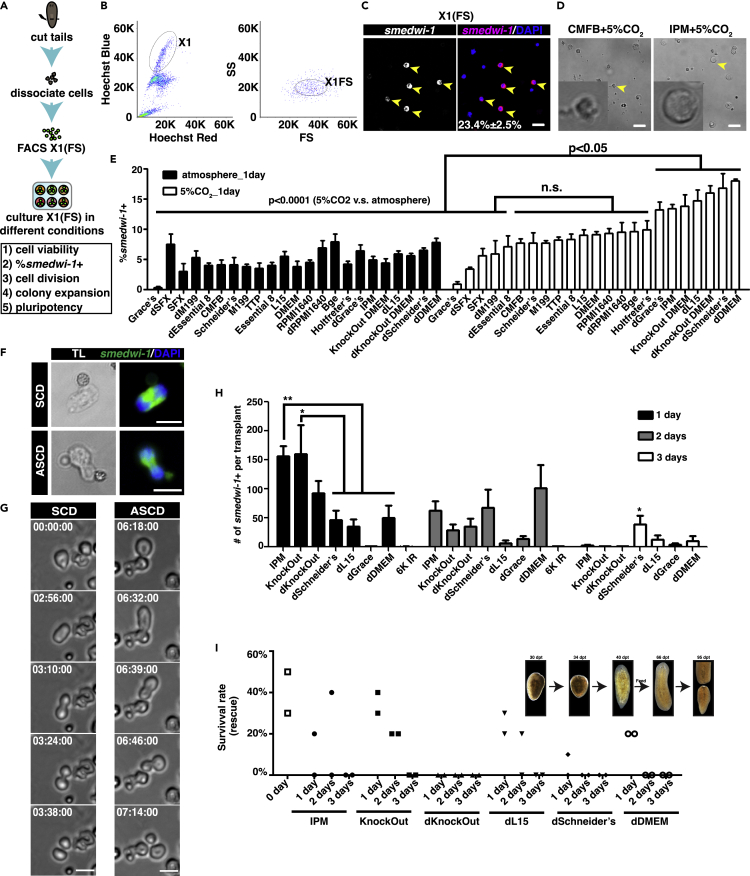
A systematic screen identifies cell culture conditions for maintaining X1(FS) neoblasts *in vitro* (A) Flowchart illustrating steps of X1(FS) cell culture and criteria used to identify best culture condition for neoblasts: cell viability, percentage of *smedwi-1*+ neoblasts (%*smedwi-1*+), cell division *in vitro*, colony expansion after transplantation, and rescue efficiency of irradiated hosts after transplantation (pluripotency). (B) Plots showing the FACS gating to sort X1(FS) cells. (C) Representative images showing *smedwi-1+* neoblasts among the sorted X1(FS) cells. Scale bar, 20 μm. X1(FS) cells consistently contain 23.4% ± 2.5% neoblasts in total DAPI + cells. Three replicates were assayed, n = 100 to 150. (D) Representative images of cell morphologies observed after 1 day of culture +5% CO_2_, including poor cell morphology in CMFB and healthy cell morphology in IPM (arrowheads). Scale bar, 20 μm. (E) Percentage of *smedwi-1+* neoblasts after 1 day of culture under indicated conditions. Significantly more *smedwi-1*+ neoblasts were maintained in seven media +5% CO_2_. Data are represented as mean ± SEM. Adjusted p values were less than 0.05 by one-way ANOVA with the Tukey test. The adjusted p-value was less than 0.001 by two-way ANOVA with the Sidak test to compare ambient atmosphere and 5% CO_2_ conditions. Three replicates were assayed, n > 500. All conditions were compared starting with the same sorted cells. (F) Representative images of dividing cells undergoing either symmetric cell division (SCD) or asymmetric cell division (ASCD). Scale bar, 10 μm. (G) Time-lapse images of dividing cells undergoing either SCD or ASCD in IPM +5% CO_2_. Scale bar, 10 μm. Both SCD and ASCD can be observed in ∼300 X1(FS) cells cultured in IPM, KnockOut DMEM, and dL15 + 5% CO_2_. (H) Number of *smedwi-1+* neoblasts in colonies formed by X1(FS) cells at 8 dpt following cultured in indicated media +5% CO_2_ for 1, 2, or 3 days. Data are represented as mean ± SEM. One-way ANOVA with the Tukey test calculated adjusted p values. ∗, 0.01 < p < 0.05; ∗∗, 0.001 < p < 0.01. Ten to twelve animals were assayed per condition. (I) Rescue rates for lethally irradiated hosts following the transplantation of X1(FS) cells cultured in the indicated media +5% CO_2_ for 1, 2, or 3 days. Each dot shows the value of the rescue rate from replicate experiments. Ten to twelve animals were assayed per condition in each experiment. The upper-right panel shows representative images of rescued hosts following the transplantation of freshly isolated X1(FS) cells, culminating in fission at 95 dpt. Scale bar, 200 μm. See also Figures S1 and S2, and Table S1.

To establish standardized neoblast culture conditions, we first used an established back-gating method for flow cytometry sorting of X1(FS) cells, which typically contain approximately 23.4% ± 2.5% neoblasts (*smedwi-1+*) (Figures 1A-1C). Testing both ambient atmosphere and 5% modified CO_2_ conditions, we systematically screened 23 different types of media, including several commercially available mammalian and insect cell culture media, previously reported formulations (e.g., IPM and TTP), and dilutions of these media that better match osmolarity suitable for planarian cells (∼120 mOsm/kg) (Table S1).^22^^,^^23^

To measure viability, cells cultured for one day were observed and stained with propidium iodide (PI), which labels the DNA of dead cells. We determined the percentage of PI negative cells by flow cytometry. Cells cultured in CMFB with or without 5% CO_2_ modification displayed irregular cell surface morphologies accompanied by sizable cellular debris, suggesting poor viability (Figure 1D). Consistent with microscopic evaluation, cells cultured in CMFB showed poor survival with or without 5% CO_2_ modification (>60% dead cells) (Figure S1A). In contrast, cells in all other conditions, such as IPM with or without 5% CO_2_, had normal morphology, suggesting high viability (Figure 1D). Among all media conditions, seventeen formulations yielded viability higher than 60% (Figure S1A). Notably, cells in Leibovitz’s L-15 medium (L15) without 5% CO_2_ extended long protrusions that were visible even after six days of culture (Figure S1B), which suggested the occurrence of neuronal differentiation, as previously observed in cultured *Caenorhabditis elegans* embryonic cells.^24^

To determine the proportion of neoblasts among total viable cells after 24 h of culture, we quantified the number of *smedwi-1*+ X1(FS) cells by fluorescent *in situ* hybridization (FISH). Notably, seven media with 5% modified CO_2_ atmosphere maintained significantly more *smedwi-1*+ neoblasts than all other conditions, including diluted (d) Grace’s medium, IPM, KnockOut DMEM, dL15 medium, dKnockOut DMEM, dSchneider’s medium, and dDMEM (Figure 1E). This result was supported by co-staining cells with *smedwi-1* and the apoptotic/dead cell marker, Annexin V, which showed no detectable co-labeling, indicating that the neoblasts were viable (Figure S1C). We next examined whether *smedwi-1+* neoblasts persisted after three days in culture using these seven media +5% CO_2_, and observed that *smedwi-1+* cells were present in all culture conditions tested here (Figure S1D). Thus, these results showed that neoblasts could be maintained for at least three days *in vitro*. We, therefore, focused on testing dGrace’s, IPM, KnockOut DMEM, dL15, dKnockOut DMEM, dSchneider’s, and dDMEM media in subsequent optimization experiments.

Next, we assessed whether cultured neoblasts could divide *in vitro.* Although we did not observe any noticeable increase in cell number, low levels of both symmetric and asymmetric neoblast divisions were observed in cells cultured for one day, as determined by cell pair size and distribution of *smedwi-1* transcripts (Figure 1F).^12^^,^^25^ Time-lapse microscopy imaging of X1(FS) cell behavior confirmed that neoblasts could divide *in vitro*. Both symmetric and asymmetric cell divisions were observed within the first 24 h in IPM, KnockOut DMEM, and dL15 medium, but not in the other four media tested (One in ∼300 X1(FS) cells) (Figure 1G and Videos S1 and S2). *PCNA* + staining assays further suggested that the proliferating cells in IPM, KnockOut DMEM, and dL15 medium were significantly more than those in CMFB, Schneider’s, and DMEM medium (Figure S1E). These results suggested that a fraction of X1(FS), *smedwi-1*+ cells can complete cell division within 24 h after isolation in culture, although we cannot exclude the possibility that these conditions only allow neoblasts in the M phase to complete the cell cycle.

Video S1. Time-lapse movie of an asymmetric cell division, related to Figure 1

Video S2. Time-lapse movie of a symmetric cell division, related to Figure 1

To determine if X1(FS) neoblasts can divide *in vivo* following *in vitro* culture, we transplanted X1(FS) cells cultured in the seven different media supplemented with 5% CO_2_ for one, two, or three days. At eight days post-transplantation (dpt), the presence or absence of *smedwi-1*+ neoblast colonies and the number of *smedwi-1*+ neoblasts in each colony were determined. All X1(FS) neoblasts cultured for one or two days efficiently proliferated *in vivo*, except for those cultured in dGrace’s medium with 5% CO_2_ (Figures 1H and S2C). By comparing the number of *smedwi-1*+ neoblasts in each transplantation, we found that X1(FS) cells cultured for one day in either IPM or KnockOut DMEM formed the largest colonies *in vivo* (Figure 1H). It was noticed that the cell number in transplanted cells that formed colonies was less than transplanted cell number. The efficiency of cell engraftment and viability after transplantation remains to be carefully determined in future studies of neoblast niches. In summary, cells grown in IPM and KnockOut DMEM performed best following one day of culture, but performed similarly to those grown in dKnockOut DMEM, dSchneider’s, dL15, and dDMEM after two days of culture. In addition, the clonogenic capacity of X1(FS) neoblasts diminished considerably following three days in culture, regardless of the medium used. These results suggested that IPM, KnockOut DMEM, dL15, dKnockOut DMEM, dSchneider’s, and dDMEM could all maintain neoblast proliferation potential for up to two days in culture in the presence of 5% CO_2_.

To evaluate the functional pluripotency of neoblasts cultured in these six media (IPM, KnockOut DMEM, dKnockOut DMEM, dL15, dSchneider’s, and dDMEM), we assessed their ability to rescue lethally irradiated hosts following bulk-cell transplantation. Transplantation of non-cultured, freshly collected X1(FS) cells resulted in the rescue of 30-50% of the lethally irradiated (6,000 rad) sexual *S. mediterranea* hosts (Figure 1I). X1(FS) cells cultured in IPM, dL15, or KnockOut DMEM for one or two days could also rescue hosts that were depleted of stem cells (Figure 1I). Genotyping PCR and restriction fragment-length polymorphism (RFLP) assays were conducted to test whether sexual hosts had been transformed into the asexual biotype following transplantation with asexual neoblasts (Figures S2D–S2G).^8^ Among the transplanted cultures, cells grown in KnockOut DMEM exhibited the highest and most robust host rescue (Figure 1I). In summary, KnockOut DMEM with 5% CO_2_ represented the most stable conditions for maintaining pluripotent neoblasts in culture for two days. Neoblasts grew in IPM and dL15 medium also retained their pluripotency for up to two days in culture, albeit with reduced rescue rates in irradiated host animals after transplantation.

### SiRNeoblasts as an alternative source of transplantable neoblasts for primary culture

**Figure 2:**
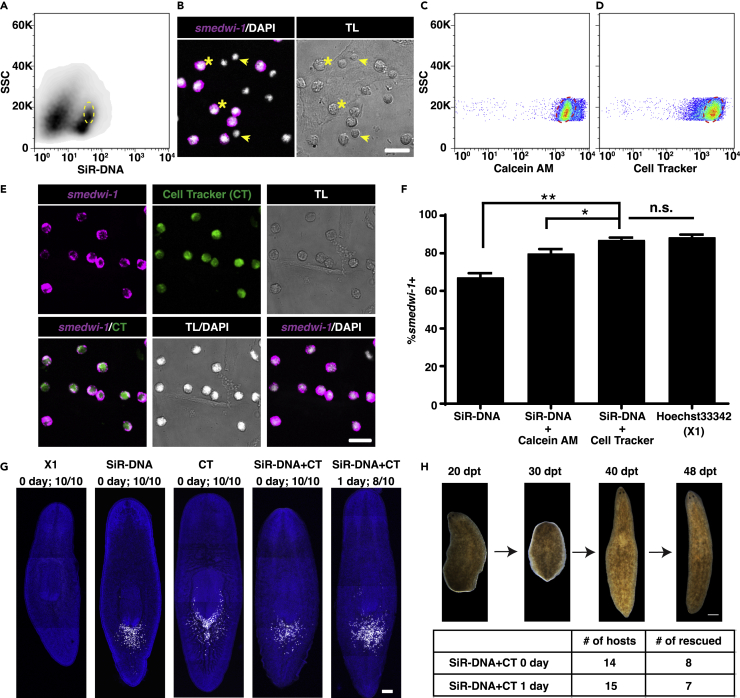
SiR-DNA plus Cell Tracker staining and cell sorting protocol enrich for clonogenic, pluripotent *smedwi-1+* neoblasts (A) Plots showing the gate used to isolate SiR-DNA + cells for *smedwi-1* ISH. (B) *smedwi-1* ISH on isolated cells from the SiR-DNA + gate shown in a. *smedwi-1*- cells (arrows) were generally smaller than *smedwi-1*+ cells (stars). Scale bar, 20 μm. (C and D) Plots showing the gates used to isolate SiR-DNA + calcein-AM + cells (C) and SiR-DNA + Cell Tracker Green + cells (D) for *smedwi-1* ISH. (E) *smedwi-1* ISH for SiR-DNA + Cell Tracker Green + neoblasts populations indicated in (D). Scale bar, 20 μm. (F) %*smedwi-1*+ neoblasts in indicated FACS isolated populations. SiR-DNA and Cell Tracker Green dual staining enrich for *smedwi-1+* neoblasts (SiRNeoblasts) comparably to the Hoechst 33342 stained X1 population. Data are represented as mean ± SEM. The Student’s *t* test was used to calculate the p values. ∗, 0.01 < p < 0.05; ∗∗, 0.001 < p < 0.01; n.s., not significant. Four random fields were assayed per condition. N > 70. (G) Representative images showing the clonogenic capacity of transplanted neoblasts obtained using different FACS isolation protocols. No noticeable difference in the colony expansion was observed among single and double dye staining populations at 7dpt. Scale bar, 200 μm. Ten animals were assayed per condition. (H) Images of a rescued host planarian and the rescue efficiency by fresh and 1-day cultured SiRNeoblasts. CT: cell tracker green. Scale bar, 500 μm. See also Figures S3 and S4.

To enrich neoblasts for culture, we tested three major types of cell-permeable DNA stains to enrich neoblasts in the G2/M cell cycle phases (DRAQ5, Vybrant DyeCycle, and SiR-DNA). The DNA stain, SiR-DNA, exhibited low cytotoxicity and resulted in ∼60% enrichment for *smedwi-1*+ neoblasts (Figures 2A, 2F, 2G, and S3A–S3C).^26^ Comparison of *smedwi-1*+ and *smedwi-1*- cell morphology in the isolated populations showed that *smedwi-1*+ cells were generally larger than *smedwi-1*- cells (Figure 2B). To discriminate between small and large cells in the SiR-DNA + population, the cytoplasmic dyes Cell Tracker Green (CT) and Calcein AM (CAM) were tested in combination with SiR-DNA for neoblast isolation (Figures 2C and 2D). This dual dye staining strategy resulted in a significant increase in neoblast enrichment, indicated by the proportion of *smedwi-1*+ cells in FISH assays (Figures 2E and 2F). In particular, SiR-DNA/CT co-staining showed comparable performance to Hoechst 33342 for enriching *smedwi-1*+ neoblasts (Figure 2F), which we designated SiRNeoblasts, as previously described.^27^

Unlike neoblasts obtained by Hoechst 33342 sorting, SiRNeoblasts proliferated and underwent colony expansion *in vivo* after transplantation into lethally irradiated planarians (Figure 2G). Importantly, no noticeable differences in colony size were observed at 7 dpt among unstained X1(FS), single (SiR-DNA)-, or double (SiR-DNA/CT)-stained populations (Figure 2G). Recently, a single-cell RNA sequencing study reported similarities between SiRNeoblasts and X1 cells, supporting that SiRNeoblasts could serve as an alternative cell source for functional studies of the neoblasts.^27^ To characterize the proportions of SiRNeoblasts in different cell cycles, we stained these cells with Hoechst 33342. Since co-staining with Hoechst and SiR-DNA blocked the SiR-DNA signal, we instead used Hoechst staining in SiR-DNA-sorted neoblasts and found that ∼17.89% of SiRNeoblasts were in the G1, ∼13.02% at S, and ∼69.09% at G2/M cell cycle phases. (Figures S3D–S3G). This reversible, low-affinity DNA staining by SiR-DNA could explain why SiRNeoblasts can proliferate after staining, while Hoechst 33342-stained X1 cells cannot.^28^

SiR-DNA staining facilitated the observation of the chromosomal separation dynamics of dividing SiRNeoblasts *in vitro* (Videos S3, S4, and S5), which confirmed the occurrence of *bona fide* cell division under the tested culture conditions. Both freshly isolated SiRNeoblasts and those cultured for one day in KnockOut DMEM with 5% CO2 could rescue lethally irradiated planarians at comparable rates to those reported for X1(FS) cells (Figure 2H). We found that ∼27.2% of SiRNeoblasts express *tgs-1,* a gene expressed in planarian pluripotent stem cells and neural progenitors,^25^^,^^29^^,^^30^ for as long as two days in KnockOut DMEM with 5% CO_2_ (Figure S4A). In addition, we observed no positive effects of co-culturing differentiated X1(FS) cells with SiRNeoblast (Figure S4B). Based on these findings, we concluded that SiR-DNA/CT dual label-based cell sorting could be used to isolate clonogenic, pluripotent neoblasts. Moreover, these isolated SiRNeoblasts can be maintained in primary culture and serve as donor cells in transplantation assays.

Video S3. Time-lapse movie of SiR-DNA-stained chromosomes in SiRNeoblasts, related to Figure 2

Video S4. Time-lapse movie of an asymmetric cell division in bright field, related to Figure 2

Video S5. Time-lapse movie of SiR-DNA-stained chromosomes in an asymmetric cell division, related to Figure 2

### Exogenous mRNA delivery by electroporation

Following the optimization of *in vitro* culture conditions to maintain neoblast pluripotency, we next tested different conditions for the delivery of exogenous (nucleic acid) molecules into neoblasts to attempt the genetic transformation of planarians. To this end, we first used dextran-FITC as a fluorescent indicator of membrane permeation to screen for the most suitable electroporation conditions of Hoechst 33342-stained neoblasts (Figure S5A). We tested 52 electroporation programs and 10 different buffers using X1 cells^31^^,^^32^ and found that dextran-FITC was most effectively delivered into neoblasts using IPM buffer with electroporation at 100–120V (Table S2 and Figures S5B–S5D). Similarly, applying this electroporation method to X1(FS) cells rather than Hoechst 33342-sorted X1 cells showed that dextran-FITC + populations could only be detected following electroporation at 110V and 120V. However, less than 6% of dextran-FITC + X1(FS) cells were *smedwi-1+* neoblasts, and virtually no *smedwi-1+* cells could be detected after one day of culture in KnockOut DMEM with 5% CO_2_ (Figure S5E). Consistent with the drastic reduction in *smedwi-1+* cell viability post-electroporation, none of the donor X1(FS) cell populations subjected to more than 100V formed colonies following transplantation into lethally irradiated donors (Figure S5F). We hypothesized that this failure was likely due to the low proportion of *smedwi-1+* neoblasts in total X1(FS) cells, which was further reduced after electroporation.

**Figure 3:**
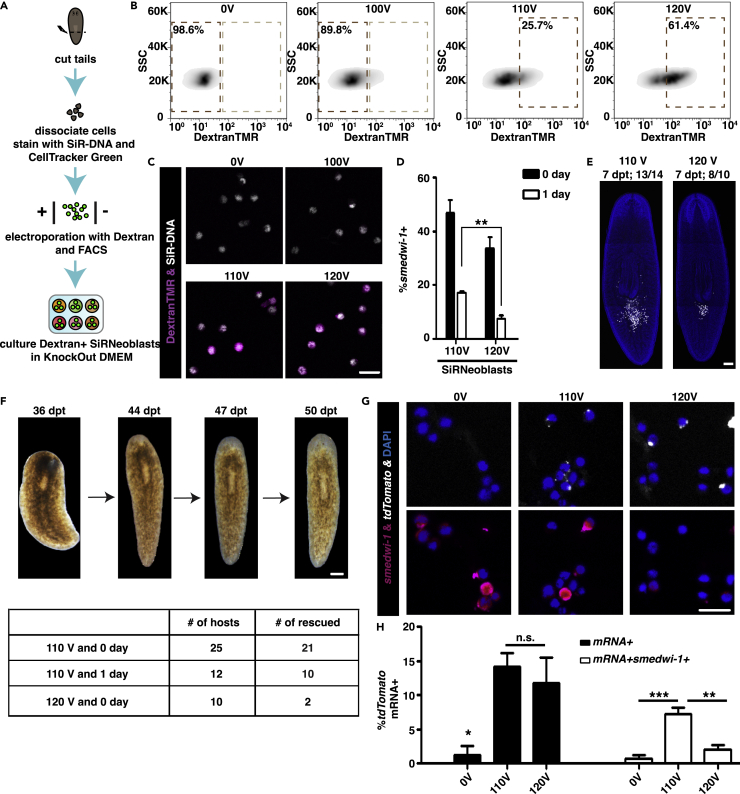
Electroporation can deliver exogenous mRNA into neoblasts (A) Flowchart presenting the steps of neoblast electroporation using SiRNeoblasts. (B) Plots showing electroporation efficiency of SiRNeoblasts at 100V, 110V, and 120V compared to 0V. (C) Neoblasts after the electroporation of Dextran-FITC showing 100% isolation of positive cells after electroporation at 110V and 120V. All SiRNeoblasts were free of Dextran-FITC without electroporation treatment. Scale bar, 20 μm. (D) The percentage of *smedwi-1*+ cells after electroporation suggests a relatively high ratio of neoblasts after electroporation using SiRNeoblasts. Four random fields were assayed per condition. Data are represented as mean ± SEM. The Student’s *t* test was used to calculate the p values. ∗∗, 0.001 < p < 0.01; (110V SiRNeoblasts vs. 120V SiRNeoblasts at 1 day). (E) Representative images showing the colony expansion of electroporated SiRNeoblasts after transplantation. Scale bar, 200 μm. N = 14 for 110V and = 10 for 120 V. (F) Images of a rescued host planarian and the rescue efficiency of electroporated SiRNeoblasts. Scale bar, 200 μm. (G) Representative images showing the mRNA signals (white dots) in cells 1 day after 110V and 120V electroporation. Scale bar, 20 μm. (H) Percentage of total cells and *smedwi-1+* cells containing mRNA 1 day after 110V and 120V electroporation. Data are represented as mean ± SEM. Adjusted p values were calculated by one-way ANOVA with the Tukey test. ∗, 0.01 < p < 0.05; ∗∗, 0.001 < p < 0.01; ∗∗∗, p < 0.001; n.s., not significant. See also Figure S5 and Table S2.

We then sought to identify the optimal electroporation conditions to retain the viability of SiRNeoblasts following the introduction of foreign genetic material (Figure 3A). Consistent with previous experiments, electroporation at 110V–120V was required for dextran-TMR internalization into SiRNeoblasts (Figures 3B and 3C). Different from that in X1(FS), *smedwi-1*+ cells were more abundant in the electroporated (110V and 120V) SiRNeoblasts compared to X1(FS) cells, and the electroporated SiRNeoblasts persisted for one day in culture (Figure 3D). In addition, the electroporated SiRNeoblasts could form colonies and rescue lethally irradiated hosts upon transplantation (Figures 3E and 3F). However, 120V electroporation resulted in SiRNeoblast rescue of relatively fewer irradiated hosts, suggesting that high voltages negatively impact SiRNeoblast viability.

To assess whether exogenous mRNA could be delivered into SiRNeoblasts by electroporation, *tdTomato* mRNA was added to the electroporation reaction along with Dextran-FITC. Dextran-FITC-positive SiRNeoblasts were sorted and cultured in KnockOut DMEM with 5% CO_2_. To confirm the successful delivery of mRNA, we probed cells via FISH at 20 h after electroporation and found detectable *tdTomato* mRNA signal in cells electroporated at either 110V or 120V (Figure 3G). However, co-staining with *smedwi-1+* revealed that not all *tdTomato* mRNA + cells retained neoblast identity in culture. The number of SiRNeoblasts positive for both *tdTomato* mRNA and *smedwi-1* expression was significantly higher after 110V electroporation than after 120V, which was a similar response to electroporation to that observed in X1 and X1(FS) cells (Figure 3H). To confirm the intracellular localization of the *tdTomato* mRNA, RNase A was used to treat cultured neoblasts at 20 h post-electroporation. The results showed that the number of *tdTomato* mRNA + cells after electroporation at 110V was comparable in the groups with and without RNase A treatment. Both groups contained significantly more *tdTomato* mRNA + cells than the matched control groups without electroporation (p < 0.01) (Figures S5G and S5H). These findings indicated that 110V electroporation was the most suitable condition for introducing exogenous, charged molecules such as RNA into neoblasts, while maintaining their viability and pluripotency.

### *Nanoluciferase* mRNA delivered by *Trans*IT can be successfully expressed in differentiating SiRNeoblasts

**Figure 4:**
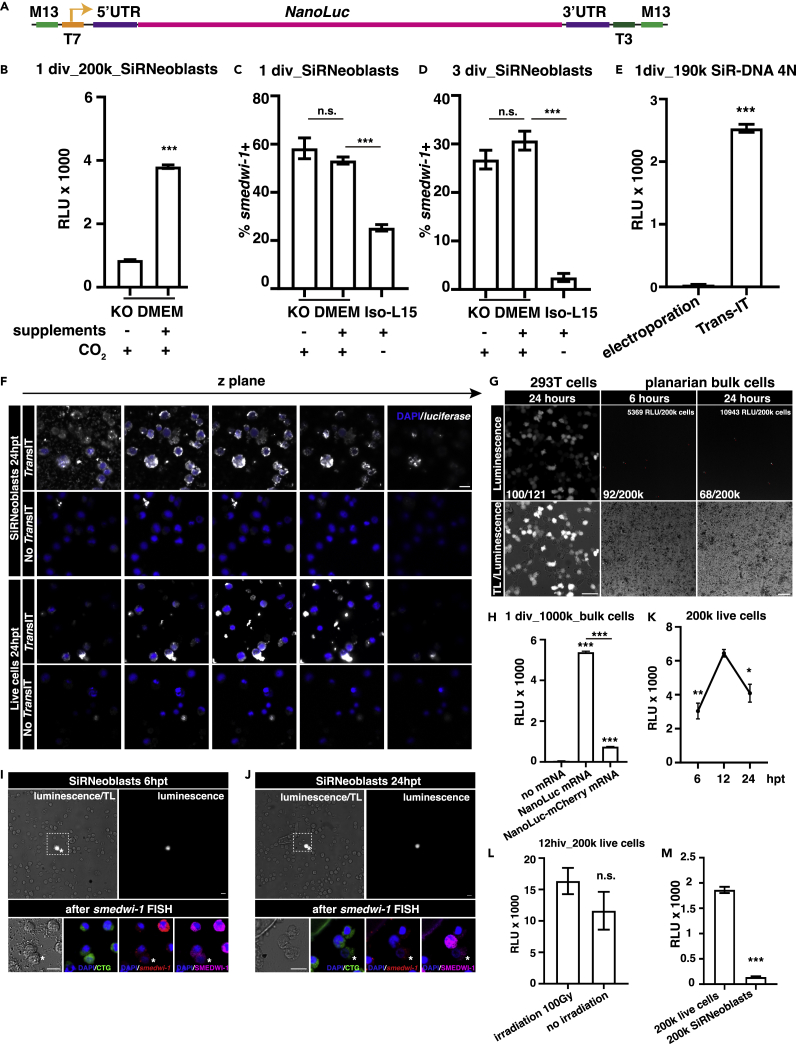
*Trans*IT can deliver exogenous mRNA into neoblasts and express NLuc in SMEDWI-1^low^ or SMEDWI-1^-^ cells (A) Adaption of the RPL15 5′UTR and -3′UTR to *NanoLuc luciferase*.^33^ (B) Expression comparison of *NanoLuc luciferase* mRNA in 200,000 SiRNeoblasts cultured with or without medium supplements after transfection by *Trans*IT. Data are represented as mean ± SEM. The Student’s *t* test was used to calculate the p values. ∗∗∗, p < 0.001; n.s., not significant. (C) The percentage of *smedwi-1*+ SiRNeoblasts in different culture conditions presence or absence of supplements or CO_2_ after 1 day of culture *in vitro* (div). Data are represented as mean ± SEM. The Student’s *t* test was used to calculate the p values. ∗∗∗, p < 0.001; n.s., not significant. (D) The percentage of *smedwi-1*+ SiRNeoblasts in different culture conditions presence or absence of supplements or CO_2_ after 3 days of culture *in vitro* (div). Data are represented as mean ± SEM. The Student’s *t* test was used to calculate the p values. ∗∗∗ p < 0.001; n.s., not significant. (E) Comparison of the efficiency of *Trans*IT and electroporation delivery methods in 190,000 SiR-DNA 4N cells transfected with *NanoLuc luciferase* mRNA. Data are represented as mean ± SEM. The Student’s *t* test was used to calculate the p values. ∗∗∗, p < 0.001. (F) Aryscan z stack of SiRNeoblasts (upper panel) and live bulk cells (lower panel) showing the *NanoLuc* mRNA localization in cells 24 h after transfection. mRNA without *Trans*IT was used as negative controls. Scale bar, 10 μm. (G) Luminescence of planarian live cells and 293T cells transfected by *Trans*IT. Scale bar, 50 μm. (H) Expression comparison of *NanoLuc* and *NanoLuc-mCherry* mRNA transfected by *Trans*IT at 24 h in live cells. Data are represented as mean ± SEM. The Student’s *t* test was used to calculate the p values. ∗∗∗, p < 0.001. (I and J) NanoLuc + cells captured at 6 h (I) and 24 h (J) post-transfection were stained with *smedwi-1* RNA probe (red) or SMEDWI-1 antibody (magenta). NLuc + cells are *smedwi-1* low or negative cells. Scale bar, 10 μm. (K) Expression comparison of *NanoLuc* mRNA in live cells transfected by *Trans*IT at 6 h, 12 h, and 24 h. Data are represented as mean ± SEM. The Student’s *t* test was used to calculate the p values. ∗, 0.01 < p < 0.05; ∗∗, 0.001 < p < 0.01. (L) Expression comparison of *NanoLuc* mRNA transfected by *Trans*IT at 12 h in live cells from wild-type and 100 Gy irradiated planarians. Data are represented as mean ± SEM. The Student’s *t* test was used to calculate the p values. n.s., p > 0.05. (M) Expression of *NanoLuc* mRNA in 200,000 planarian live cells or SiRNeoblasts transfected by *Trans*IT in KnockOut DMEM + supplements +5%CO_2_. Data are represented as mean ± SEM. The Student’s *t* test was used to calculate the p values. ∗∗∗, p < 0.001. See also Figures S6 and S7, and Table S3.

Unfortunately, tdTomato expression was undetectable by either microscopy or antibody staining in cultured neoblasts. A recent study reported that *Nanoluciferase* (*NanoLuc*) mRNA could be expressed in somatic planarian cells through Viromer or *Trans*IT transfection.^33^ We, therefore, suspected that the high sensitivity and low autofluorescence background of the NanoLuc reporter could provide a tractable approach for visualizing neoblast transgene expression. To confirm that *NanoLuc* was indeed translated in cultured SiRNeoblasts, we transfected SiRNeoblasts with *NanoLuc* mRNA using the *Trans*IT system (Figure 4A). Culture medium supplements (sodium pyruvate, vitamin, and amino acids, see Table S1) were also included in the modified KnockOut DMEM to determine whether they could enhance the mRNA expression.^33^ We found that NanoLuc expression levels were higher in SiRNeoblasts cultured in modified KnockOut DMEM with 5% CO_2_ modified atmosphere than in cells grown under the same conditions without supplements (Figure 4B). To confirm that neoblasts could also be maintained in modified KnockOut DMEM with 5% CO_2_, SiRNeoblasts were stained and their proportion was compared to that of *smedwi-1*+ cells after one or three days of culture in modified KnockOut DMEM with 5% CO_2_, KnockOut DMEM with 5% CO_2_, and Iso-L15 under ambient conditions. The results showed that SiRNeoblasts could be maintained in modified Knockout DMEM as well as the Knockout DMEM, but not the Iso-L15 (Figures 4C and 4D), which combined with earlier findings that higher CO_2_ was required for consistently high SiRNeoblast activity (Figure 1E), led us to use modified KnockOut DMEM with 5% CO_2_ modification in subsequent experiments.

After delivering the *NanoLuc* mRNA into neoblasts by the two different methods (*Trans*IT transfection and electroporation), we found that the NanoLuc signal was only detectable following *Trans*IT transfection (Figure 4E). We next sought to compare the efficiency of mRNA delivery between *Trans*IT and electroporation. Electroporation at 110V delivered *NanoLuc* mRNA into 11.22% of neoblasts, similar to that of *tdTomato* mRNA (Figure S5I). In contrast, *Trans*IT transfection delivered *NanoLuc* mRNA into ∼100% of cells tested (Figures 4F and S6A). Also, the percentage of *NanoLuc* mRNA + cells after RNase A treatment was indistinguishable from those without RNase A treatment (∼100%) (Figure S6A). Furthermore, compared with the fluorescent intensity of *NanoLuc* mRNA with poly-A tail, the signal was obviously weaker within SiRNeoblasts after the transfection of *NanoLuc* mRNA without a poly-A tail (Figure S6B). These results indicated that *Trans*IT can indeed deliver *NanoLuc* mRNA into SiRNeoblasts with higher efficiency than electroporation.

To confirm this methodology with other mRNAs, we used *Trans*IT to deliver mRNA encoding *NanoLuc*, *smed-histone3.3-2xflag*, *mCherry*, or *NanoLuc-mCherry*. Surprisingly, none of these proteins were detectable by Western blot (Figures S6C–S6F). In addition, immunofluorescence staining of transfected SiRNeoblasts could not provide a definitively positive signal because of autofluorescence or non-specific antibody binding in planarian cells (Figures S6G–S6I), although the nuclear-localized staining signals were only captured in *smed-histone3.3-2xflag* mRNA transfected cells (Figure S6I). Using chemiluminescence signal imaging, we observed that the ratio of NanoLuc + cells was much lower in live planarian cells (68/200k) than in 293T cells (100/121) (Figure 4G). Since luciferase chemiluminescence assays have reportedly extremely high sensitivity for detecting signals from a small proportion of positive cells, this meager ratio of NanoLuc + cells may explain the success of the high sensitive chemiluminescence, but the absence of immunofluorescent signals. However, *NanoLuc-mCherry* transcripts exhibited a measurably lower signal likely due to a relatively longer coding sequence for reduced transfection efficiency, which suggested that the signal was indeed due to *NanoLuc* transcript expression (Figure 4H).

We next checked whether NanoLuc + cells retained their *smedwi-1*+ phenotype to further investigate the potential low translation efficiency. The results clearly showed that all the NanoLuc + cells in our experiments were neither *smedwi-1*+ nor SMEDWI-1+ (Figures 4I, 4J, S6J, and S7) (note: CTG + indicates whether cells were alive at the time of fixation). Since silencing mechanisms to suppress exogenous gene expression have long been suspected as a confounding factor in neoblast transformation experiments, this phenomenon strongly suggested the function of an unknown mechanism in silencing the expression of exogenous mRNA in neoblasts. We compared NanoLuc levels in SiRNeoblasts with that in bulk live cells to test this hypothesis. After confirming the temporal dynamics of NanoLuc expression by chemiluminescence (Figure 4K), we also compared the signal between live bulk cells from non-irradiated and lethally irradiated planarian populations. The results indicated that live bulk cells exhibited considerably higher NanoLuc signal than that in SiRNeoblasts, even after the depletion of neoblasts by lethal irradiation (Figures 4L, 4M, and S6K), which was consistent with the findings of Hall et al.^33^ This result indicated that the NanoLuc signal was derived almost exclusively from somatic cells, and further suggested that planarian neoblasts harbored a means of preventing exogenous nucleic acids before differentiation.

## Discussion

The inability to genetically transform planarians has posed a long-standing obstacle to researching this otherwise highly versatile model for pluripotency and whole-body regeneration. The primary technical limitations underlying this obstacle involve determining the optimal culture conditions for maintaining pluripotent neoblasts and identifying an effective means for delivering exogenous nucleic acids into these cells. The cell culture system we have developed in this work resolves the former problem and enables further testing of strategies for exogenous material delivery, such as fluorescence-conjugated dextrans and mRNA, to ultimately demonstrate the translation of introduced mRNAs. Our method establishes the requisite foundation for developing transgenic and genome editing techniques in planarians to enable exciting new systematic investigations of naturally occurring pluripotent adult stem cell populations.

First, the use of SiRNeoblasts ensures the purity and viability of neoblasts, thus allowing relative ease in screening transgene delivery strategies. Moreover, we propose that positively charged polymers, not limited to *Trans*IT, could be used to deliver larger molecules and genome-editing tools with higher transfection efficiency and a higher likelihood of obtaining transgenic animals.

Second, the low efficiency of transfection and translation may also be due to cultured cells' relatively decreased metabolic activity. The observed enhancement of *NanoLuc* mRNA translation following the addition of supplements suggested that the uptake and translation of mRNA depended on meeting metabolic requirements in cultured neoblasts. The cell culture platform described here provides a reliable approach for identifying nutrient requirements by comparing cultured neoblasts with *in vivo* neoblasts. Adding supplements to culture media can also optimize long-term culture systems and cell lines, enabling downstream research of transformation techniques and functional validation of other genetic manipulations (e.g., CRISPR RNPs for genome editing) in cultured cells. Meanwhile, the mechanism of why neoblast pluripotency maintenance requires 5% CO_2_ has remained to be investigated.

Third, given that neoblasts are the *de facto* units of selection in planarians and that the viability of these animals heavily depends on their proper function and viability, it is logical that these cells have evolved robust molecular mechanisms to protect their genome from disruption by foreign nucleic acids. In the current study, we did observe that NanoLuc + cells were neither *smedwi-1*+ nor SMEDWI-1+, consistent with this hypothesis. Further experiments are necessary for definitive evidence supporting or refuting this hypothesis.

In summary, we describe a FACS isolation strategy and primary cell culture conditions for maintaining clonogenic, pluripotent neoblasts *in vitro* in short-term compatible with transplantation, repopulation, and rescue of lethally irradiated hosts. In addition, we demonstrate the successful introduction of exogenous mRNAs into neoblasts. Although further optimization is needed, this finding represents a significant technical milestone in developing protocols for generating transgenic planarians. Together with the findings of Hall et al., these results show that highly sensitive NanoLuc reporters can be robustly expressed in planarian cells. Interestingly, we found that cells labeled with traditional Hoechst 33342 staining, including X1, X2, and Xins, could not express NanoLuc, which supported the use of alternative means of obtaining neoblasts, such as SiRNeoblasts and CRNeoblasts, in further efforts to optimize neoblast culture conditions (Figure S6K). Our results also strongly suggest the presence of a long-suspected silencing mechanism in planarian neoblasts for suppressing exogenous gene expression, thus opening an avenue for further study into this potential mode of gene suppression and enabling the genetic transformation of regenerative planarian populations.

### Limitations of the study

The cell culture condition we have developed in this study does not allow the long-term proliferation of neoblasts. Better cell culture conditions to solve this problem will be investigated in future studies. The current cell culture condition also did not show the detection of fluorescent protein, such as tdTomato and mCherry. The accomplishment of neoblast long-term culture may also provide a solution to increase the expression level of exogenously delivered genes and to achieve cell transformation for planarians.

## STAR★Methods

### Key resources table

**Table:** 

REAGENT or RESOURCE	SOURCE	IDENTIFIER
**Antibodies**		

Anti-digoxigenin-POD	Roche	Cat# 11207733910, AB_514500
Anti-fluorescein-POD	Roche	Cat# 11426346910, AB_840257
Anti-phospho-Histone H3 (Ser10) (H3P) antibody	Abcam	Cat# ab32107, RRID:AB_732930
Rabbit polyclonal RFP antibody	MBL	Cat# PM005, RRID:AB_591279
Mouse monoclonal Flag antibody clone M2	Sigma	Cat# F1804, RRID:AB_262044
NanoLuc antibody	Promega	Cat# N7000
Anti-tubulin antibody	GenScript	Cat# A01410, RRID:AB_1968943
Alexa 555-conjugated goat anti-rabbit antibody	Abcam	Cat# ab150086, RRID:AB_2890032
Alexa 555-conjugated goat anti-mouse antibody	Abcam	Cat# ab150118, RRID:AB_2714033
Alexa 647-conjugated goat anti-rabbit antibody	Abcam	Cat# ab150083, RRID:AB_2714032
Alexa 647-conjugated goat anti-mouse antibody	Abcam	Cat# ab150119, RRID:AB_2811129
Goat Anti-Mouse IgG antibody (H + L) HRP	GenScript	Cat# A00160, RRID:AB_1968937
Goat Anti-Rabbit IgG antibody (H + L) HRP	GenScript	Cat# A00098, RRID:AB_1968815

**Bacterial and virus strains**		

*Escherichia coli* DH5α	N/A	N/A

**Chemicals, peptides, and recombinant proteins**		

KnockOut DMEM	ThermoFisher	Cat# 10829018
Schneiders’s Drosophilia Medium	ThermoFisher	Cat# 21720024
Dulbecco’s Modified Eagle Medium	ThermoFisher	Cat# 11995073
Essential 8 Medium	ThermoFisher	Cat# A1517001
Grace’s Insect Media	ThermoFisher	Cat# 11605094
Leibovitz’s L-15 Medium	Hyclone	Cat# SH30525.01
Medium 199	Gibco	Cat# 12350039
RPMI 1640	Corning	Cat# 10-040-CV
SFX-Insect Cell Culture Media	Hyclone	Cat# SH30278.01
*Biomphalaria glabrata* embryonic cell Medium	N/A	See Table S1
Calcium magnesium-free Medium, BSA	N/A	See Table S1
Isotonic Planarian Medium (IPM)	N/A	See Table S1
Teshirogi and Tohya Planarian Medium (TTP)	N/A	See Table S1
Fetal Bovine Serum	Sigma	Cat# F4135
Penicillin Streptomycin	Gibco	Cat# 15140-122
100x MEM Vitamin Solution	Gibco	Cat# 11120-052
100x MEM Non-Essential Amino Acid	Gibco	Cat# 11140-050
100 mM Sodium Pyruvate	Gibco	Cat# 11360-070
OPTI MEM	Genom	Cat# GNM226000-1
Poly-D-lysine	BD Biosciences	Cat# 354210
Gentamicin	Gemini	Cat# 400-100P
RNase A	Vazyme	Cat# DC201-1
Formaldehyde	Sigma	Cat# F8775
Paraformaldehyde	Electron Microscopy Sciences	Cat# 15710
RIPA	Genstar	Cat# E122-01
Protease inhibitor cocktail	MCE	Cat# HY-K0010
Prolong gold antifade reagent	ThermoFisher	Cat# P36934
10x PBS (for cell sorting)	Solarbio	Cat# P1022

**Critical commercial assays**		

Tyramide-conjugated Cy3	Sigma-Aldrich	Cat# PA13101
Tyramide-conjugated Cy5	Sigma-Aldrich	Cat# PA15101
Hoechst 33342	ThermoFisher	Cat# H3570
DRAQ5	ThermoFisher	Cat# 62254
Vybrant DyeCycle Ruby stain	ThermoFisher	Cat# V10309
SiR-DNA	Cytoskeleton	Cat# CY-SC007
CellTracker Green CMFDA Dye	ThermoFisher	Cat# C7025
Annexin V FITC Conjugate	BioLegend	Cat# 640905
Dextran-FITC	ThermoFisher	Cat# D3306
DAPI	ThermoFisher	Cat# D3571
mMESSAGE mMACHINE T7 ultra kit	ThermoFisher	Cat# AM1345
MEGAClear™ kit	ThermoFisher	Cat# AM1908
Cell Line Optimization 4D-Nucleofector X Kit	Lonza	Cat# V4XC-9064
Primary Cell Optimization 4D-Nucleofector X Kit	Lonza	Cat# V4XP-9096
TransIT-mRNA Transfection Kit	ThermoFisher	Cat# MIR 2225
Nano-Glo Dual-Luciferase Reporter assay	Promega	Cat# N1610
Nano-Glo luciferase assay	Promega	Cat# N1110
5x Phusion Reaction Buffer	NEB	Cat#B0518S
dNTP mix	Vazyme	Cat#P301-AA
50 mM MgCl2	NEB	Cat#B0510A
Phusion High-Fidelity DNA Polymerase	NEB	Cat#M0530L
T7 RNA Polymerase	Promega	Cat#P207E
StarPrep Gel Extraction Kit	GenStar	Cat# D205-04
FsatPure Plasmid Mini Kit	Vazyme	Cat# DC201-01
MicroSpin G-50 Columns	Cytiva	Cat# 27533002

**Experimental models: Cell lines**		

Human: 293T cell line	N/A	N/A

**Experimental models: Organisms/strains**		

*Schmidtea mediterranea*, asexual	N/A	CIW4
*Schmidtea mediterranea*, sexual	N/A	S2F1L3F2

**Oligonucleotides**		

Primers for *tdTomato* mRNA template(fw): CAGATTAATACGACTCACTATAGG	This paper	N/A
Primers for *tdTomato* mRNA template(rev): ACTGATAATTAACCCTCACTAAAG	This paper	N/A
Primers for *NanoLuc* luciferase mRNA template(fw): CAGATTAATACGACTCACTATAGG	This paper	N/A
Primers for *NanoLuc* luciferase mRNA template(rev): ACTGATAATTAACCCTCACTAAAG	This paper	N/A

**Recombinant DNA**		

pcDNA3.1(+)::tdTomato	This paper	N/A
RPL15::mCherry	This paper	N/A
RPL15::histone3.3	This paper	N/A
RPL15::NanoLuc	This paper	N/A

**Software and algorithms**		

ImageJ (FIJI)	Schindelin et al., 2012	https://fiji.sc
GraphPad Prism 6.0	GraphPad Software, La Jolla California USA	https://www.graphpad.com/scientificsoftware/prism/
FlowJo_V10	Becton, Dickinson and Company; 2021	https://www.flowjo.com/solutions/flowjo

**Other**		

Eppendorf FemtoJet	Eppendorf	N/A
Borosilicate glass microcapillary	Sutter	Cat# B100-75-15
Celigo imaging cell cytometer	Celigo	N/A
FACAria Fusion SORP	BD	N/A
BTX ECM830 electroporator	BTX	N/A
Lonza 4D electroporator	Lonza	N/A
VarioskanTM LUX multimode microplate reader	ThermoFisher	Cat# N16044
Andor iKon-M 934 CCD	Andor	N/A
DMi8 inverted microscopes	Leica	N/A
C2Si confocal microscope	Nikon	N/A
LSM 800 with Airyscan	Zeiss	N/A
Eclipse TE2000-E	Nikon	N/A
SurePAGE, Bis-Tris, 4-20% gel	GenScript	Cat# M00655
6-cm dish	MatTek	Cat# P35G-1.5-14-C
24-well plate	MatTek	Cat# P24G-1.5-13-F
96-well plate	WHB	Cat# WHB-96
384-well plate	Greiner bio-one	Cat# 781090
96-well black plate	ThermoFisher	Cat# 446471

### Resource availability

#### Lead contact

Further information and requests for resources and reagents should be directed to and will be fulfilled by the Lead Contact, Alejandro Sánchez Alvarado (asa@stowers.org).

#### Materials availability

All reagents are available from the lead contact upon reasonable request.

### Experimental model and subject details

#### Planarian care and irradiation treatment

Asexual (Clone CIW4) and sexual (Clone S2F1L3F2) strains of *Schmidtea mediterranea* were maintained in Montjuïc water at 20°C.^34^ Animals were starved for 7–14 days before each experiment. Animals exposed to 6,000 rads of γ rays were used as transplant hosts.^8^ After transplantation, hosts were maintained in Montjuïc water with 50 μg/mL of Gentamicin (GEMINI, 400-100P). For transplant rescue experiments, host animals were kept in 3.5 cm Petri dishes (1 worm/dish), and Montjuïc water was changed every 2–3 days.

### Method details

#### Cell staining

Tails from planarians (>8 mm in length) were chopped into small pieces with a blade in Calcium Magnesium free buffer with 1% Bovine Serum Albumin (CMFB, Recipe in Table S1). The tissue pieces were then dissociated in CMFB for 20–30 min (no more than 30 min) with vigorous pipetting every 3–5 min until there were no visible tissue pieces. Dissociated cells were centrifuged at 290 × g for 10 min at 4°C after being filtered through a 70 μm strainer. Cells were then resuspended in Isotonic Planarian Medium (IPM, Recipe in Table S1) with 10% Fetal Bovine Serum at 3 × 10^6^ cell density for either Hoechst 33342 or SiR-DNA + CellTracker Green staining. To get X1 cells, dissociated cells in IPM (10% FBS) were stained with Hoechst 33342 (0.4 mg/mL, ThermoFisher Scientific, H3570) for 45 min. To enrich neoblasts, DRAQ5 (ThermoFisher Scientific, 62254, 5 μM), Vybrant DyeCycle Ruby stain (ThermoFisher Scientific, V10309, 10 μM), and SiR-DNA (1 μM, Cytoskeleton Inc., CY-SC007) were compared. To obtain SiRNeoblasts, dissociated cells were stained with SiR-DNA (1 μM, Cytoskeleton Inc., CY-SC007) for 1 h and then CellTracker Green CMFDA Dye (2.5 μg/mL, Thermo Fisher Technologies, C7025) for 10 min. SiR-DNA and CellTracker Green CMFDA Dye were resolved in DMSO. The final concentration of DMSO should be less than 0.1%. All staining was performed in the dark at room temperature. Tubes should be gently flicked every 5 min during the staining process to prevent cell aggregation leading to poor staining. Stained cells were centrifuged at 290 × g for 10 min to remove dyes and resuspended with IPM (10% FBS). DAPI was added to the cell suspension at 1 μg/mL before loading on the cell sorter.

#### Flow cytometry and cell collection

The Influx or BD FACS Aria (FACAria Fusion SORP) cell sorter was set with a 100 μm tip/nozzle and multiple lasers (355, 488, 561, and 647 nm) in the collection mode. 0.4 X PBS was prepared as the sheath liquid by diluting 10 X PBS (Solarbio, P1022) with ultrapure water. The cells were sorted into 15 mL tubes at a flow rate of no more than 2. The number of sorted cells was set to meet the experimental requirements.

#### FACS of X1 and X1(FS)

X1(FS) cells were collected according to a pre-stained X1 population.^8^^,^^31^ The gate in the BUV737 (355 nm, 740/35)/Hoechst 33342 (355 nm, 515/30) plot was set for the X1 population. To gate the X1(FS) cells, the X1 population from a control sample was used to define the forward scatter/side scatter gate.

#### FACS of SiRNeoblasts

SiRNeoblasts were collected after staining planarian cells with SiR-DNA and CellTracker Green CMFDA.^27^ The gate in APC (647 nm, 670/30)/SSC plot was used to enrich the 4N cells. Populations with fluorescence intensity of FITC channel (488 nm, 530/30) within the top 60%–65% were selected as SiRNeoblasts. Sorted cells were centrifuged at 290 × g for 10 min to remove the collection solution and resuspended with culture medium for further experiments.

#### Enrichment of bulk live cells

Dissociated cells without staining were filtered once through a 70-micron filter and twice through a 35-micron filter. Bulk cells in the flowthrough were centrifuged at 290 × g for 10 min to remove the collection solution and resuspended with culture medium for further experiments.

#### Cell culture

Dishes and multi-well plates were pre-coated with poly-D-lysine (50 μg/mL, BD Biosciences). If not specified, 1 X 10^4^ X1(FS) cells or SiRNeoblasts were cultured in 50 μL indicated culture medium containing 5% Fetal Bovine Serum (Sigma-Aldrich, F4135) per well in 384-well plates (Greiner bio-one, 781090) at 22°C with 5% CO_2_ or ambient atmosphere.

For time-lapse imaging experiments, 6 X 10^5^ X1(FS) cells were pre-loaded in the center of a PDL-coated MatTek dish with 100 μL medium for 30 min and then added in either 5 mL of the indicated culture medium per well of 6 cm dishes (MatTek, P35G-1.5-14-C) or 1 mL of the indicated culture medium per well of 24-well plates (MatTek, P24G-1.5-13-F).

For transfection experiments, if not specified, 2 × 10^5^ bulk cells, sorted live cells, or SiRNeoblasts were cultured in 96-well plates with 225 μL medium (with or without supplements) per well. Supplements containing 100 X MEM Vitamin Solution (Gibco, 11120-052), 100 X MEM Non-Essential Amino Acid (Gibco, 11140-050), 100 mM Sodium Pyruvate (Gibco, 11360-070), and Penicillin Streptomycin (Gibco, 15140-122) were added to the basal medium at the 1:100 dilution.

#### *In situ* hybridization and antibody staining

For ISH on cultured cells, cell culture plates were centrifuged in an Eppendorf horizontal centrifuge (Centrifuge 5810 R) at 300 × *g* for 3 min. Cells were fixed with 3.7% formaldehyde (Sigma-Aldrich, F8775) or 4% paraformaldehyde (Electron Microscopy Sciences, 15710) for 20 min. After washing with 1× PBS, cells were hybridized with riboprobes at 56°C for at least 15 h. After washing with 2× SSC and 0.2× SSC, cells were incubated with anti-digoxigenin-POD (Roche Diagnostics, 11207733910) or anti-fluorescein-POD (Roche Diagnostics, 11426346910) at room temperature for 2 h. After washing with 1× PBS/0.3% Triton X-100, the signal was developed using tyramide-conjugated Cy3 (Sigma-Aldrich, PA13101) or Cy5 (Sigma-Aldrich, PA15101).

Anti-phospho-Histone H3 (Ser10) (H3P) antibody (1:1,000, Abcam, ab32107) and Alexa 555-conjugated goat anti-rabbit secondary antibodies (1:1,000, Abcam, ab150086) were used to stain proliferating cells at the G2/M phase of the cell cycle.

To stain *smedwi-1* mRNA and SMEDWI-1 protein in NanoLuc + cells, NanoLuc + cells in the well were firstly located through luminescence imaging. *Smedwi-1 in situ* hybridization and SMEDWI-1 antibody (gift from Jochen Rink, 1:5000) staining were performed after fixation of cells in the plate. Finally, NanoLuc + cells were found through their location in the well to image the staining of *smedwi-1* mRNA or SMEDWI-1 protein.

#### Annexin V staining

Fifty microliters of cultured cells were resuspended and stained with 2.5 μL of Annexin V FITC Conjugate (BioLegend, 640,905) at room temperature for 15 min. After washing twice with IPM +10%FBS, cells were subjected to *smedwi-1* ISH. After that, anti-fluorescein-POD (Roche Diagnostics, 11426346910) was used to stain Annexin V for apoptotic and dead cells detection.

#### Cell transplantation

X1(FS) cells collected by flow cytometry were transplanted into irradiated hosts (6,000 rads).^8^ Approximately 1 μL of an X1(FS) cell suspension (5,000 cells/μL) was injected into either the post-pharyngeal midline (of asexual CIW4 hosts) or the post-gonopore midline (of sexual S2F1L3F2 hosts) at 0.75–1.0 psi (Eppendorf FemtoJet) using a borosilicate glass microcapillary (Sutter Instrument Co., B100-75-15). Serial cell dilution experiments indicated that bulk cell transplantation of 1 × 10^3^ freshly collected X1(FS) cells resulted in colony expansion in ≥80% hosts (Figures S2A and S2B). Considering the rate of ∼10% cell death in culture, we cultured 5 X 10^4^ X1(FS) cells for each test condition to ensure that sufficient viable cells were available at the time of transplant.

#### mRNA synthesis and electroporation

According to the protocol, the capped mRNA with poly(A) tail was transcribed *in vitro* via mMESSAGE mMACHINE T7 ultra kit (ThermoFisher Scientific, AM1345). tdTomato mRNA was transcribed from the linearized plasmid pcDNA3.1 (+)-tdTomato. The PCR product used as a template was amplified by primers 5′-CAGATTAATACGACTCACTATAGG-3′ and 5′-ACTGATAATTAACCCTCACTAAAG-3′. The mRNA was purified by MEGAClearTM kit (ThermoFisher Scientific, AM1908).

Cells from four tail fragments of 8–10 mm planarians were suspended in 20 μL electroporation buffers following Hoechst 33342 staining to screen electroporation conditions. 20 μg Dextran-FITC (ThermoFisher Scientific, D3306) were mixed with cells and loaded into a 1 mm electroporation cuvette for BTX ECM830 electroporator or a 12-well electroporation strip for Lonza 4D electroporator. The buffer SE, SG, SF, P1-5 were electroporation buffers in Lonza Cell Line and Primary Cell 4D-Nucleofector Optimization kits (V4XC-9064 and V4XP-9096). Cell viability and electroporation efficiency were assessed using an Influx sorter.

For exogenous mRNA electroporation, ∼1x10^8^ cells were suspended in 50 μL IPM following SiR-DNA staining. 50 μg Dextran-FITC and ∼5 μg mRNA were mixed with cells and loaded into a 1 mm electroporation cuvette. BTX ECM830 electroporator was used to apply a 110 V and 1-millisecond square wave pulse to deliver dextran-FITC and mRNA into planarian cells. Dextran-FITC + SiRNeoblasts were purified using an Influx sorter and cultured in KnockOut DMEM +5%FBS. Culture cells were washed with medium and treated with 1% (V/V) RNase A (Vazyme, DC201-1) for 30min before fixation and ISH.

#### Microscopy and time-lapse imaging

The Celigo imaging cell cytometer (Celigo, Inc.) and the Falcon 700 confocal microscope were used to take pictures of X1(FS) and SiRNeoblasts following ISH. Celigo or ImageJ software was used for quantitative analyses. A Nikon Eclipse TE2000-E equipped with Perfect Focus and a Plan Fluor ELWD 20X/0.45 NA Ph1 objective was used to perform time-lapse imaging of cultured cells. Micro-manager was used to control the microscope and Hamamatsu Orca R2 CCD.^35^ Multiple positions were acquired at 5-min intervals for 24–48 h. *In situ* hybridization samples were imaged with a Nikon Eclipse Ti equipped with a Yokogawa W1 spinning disk head and a Prior PLW20 Well Plate loader. Several slides were prepared at once and then loaded and processed automatically using a combination of Nikon Elements Jobs for all robot and microscope control and Fiji for object-finding and segmentation. Slides were imaged at low magnification, and objects were identified before re-imaging tiled z-stacks using a Plan Apo 10X 0.5NA air objective. Tiled images were stitched, projected, and *smedwi-1+* puncta were counted using custom macros and plugins in Fiji.

#### Exogenous mRNA chemical transfection

For the transfection of planarian live cells, bulk cells or SiRNeoblasts, 1.5 μg mRNA was mixed with 2 μL mRNA Boost Reagent and 1 μL *Trans*IT-mRNA Reagent (*Trans*IT-mRNA Transfection Kit, MIR 2225) in Opti-MEM (Genom, GNM226000-1). Finally, the mix was diluted into 225 μL culture medium after incubating for 3 min at room temperature. Cells were centrifuged at 290 × g for 10 min and then resuspended by 250 μL transfection mixture and incubated in 96-well plate (WHB-96) for 6h, 12h or 24 h. The SiRNeoblasts were resuspended for luminescence detection, western blot, or ISH at indicated time points. For RNase treatment, 5 × 10^4^ cultured cells were treated with 5% (V/V) RNase A (Vazyme, DC201-1) for 2 h before fixation and ISH. For 293T cells, 0.09 μg mRNA was used to transfect as protocol recommended in 96-well plate.

#### Luminescence detection assay

The Nano-Glo Dual-Luciferase Reporter assay (Promega, N1610) was used to detect the expression of *RPL15-NLuc* mRNA. The cells suspended in 50 μL culture medium were mixed well with an equal volume of ONE-Glo EX Reagent and then transferred to C8 black Nunc 96-well plate (ThermoFisher Scientific, 446,473) at room temperature. After incubation for 3 min, the lysate was mixed with 50 μL Stop & Glo reagent (ratio of substrate to buffer in 1:100) for luminescence detection. The Varioskan™ LUX multimode microplate reader (ThermoFisher Scientific, N16044) was used to measure the relative light unit of luminescence signals. The luminescence of each well was recorded three times with 1000-ms measuring time.

#### Luminescence imaging

The Leica DMi8 microscopy and Andor iKon-M 934 CCD camera were used to take pictures of luminescence from planarian cells and 293T cells with a 20x air objective (Leica, 506521). The 200,000 planarian live cells were resuspended in 50 μL culture medium and transferred to a glass-bottom dish (NEST, 801002) for imaging. The luminescence was measured after directly adding 1 μL Nano-Glo luciferase assay substrate (Promega, N1110) to cells. The exposure time was set to 20 s for luminescence imaging, and 1 s for bright field imaging.

#### Western blot

The 1000,000 planarian live cells were sorted out for transfection and protein extraction. Each protein sample of cells in 96- well plates were collected in PCR tube 24 h post-transfection and homogenized in 25 μL RIPA (RIPA lysis buffer (Genstar, E122-01), 1 mM PMSF, 10 mM DTT, 1X protease inhibitor cocktail (MCE, HY-K0010)). All the protein samples were loaded for immunoblotting. The antibodies used were as follows, rabbit polyclonal RFP antibody (MBL, PM005), mouse monoclonal Flag antibody clone M2 (Sigma, F1804), NanoLuc antibody (Promega, N7000), α-tubulin antibody (GenScript, A01410), goat anti-mouse IgG antibody (H + L) HRP (GenScript, A00160), goat anti-rabbit IgG antibody (H + L) HRP (GenScript, A00098). The primary antibodies were used in 1:1000 dilution, and secondary antibodies in 1:20,000.

#### Antibody immunofluorescence staining in cells

The 293T cells were cultured at coverslips and transfected with *RPL15-mCherry_4 and RPL-histone3.3* mRNA by *Trans*IT, and fixed by 4% PFA for antibody immunofluorescence staining after 24 h of transfection. The samples were incubated in primary antibodies (1:500) for 3 h and secondary antibodies conjugated with Alexa Fluor 647 (1:500) for 2 h, with 15 min DAPI staining before being mounted in Prolong gold antifade reagent (ThermoFisher Scientific, P36934). For SiRNeoblasts, 20,000 cells were sorted out for each group and transfected with *RPL15-mCherry_4 and RPL-histone3.3* mRNA by *Trans*IT. At 24 hpt, following fixed by 4% FA in 0.4X PBS twice for 10 min, incubated in Hybe at 56°C for 2 h and blocked by 10% Horse serum in PBSTx0.1% at room temperature for 30 min, the cells were stained with primary antibodies (1:500) and secondary antibodies conjugated with Alexa Fluor 555 (1:500) for 2 h, respectively. The antibodies included rabbit polyclonal mCherry antibody (MBL, PM005), mouse monoclonal Flag antibody clone M2 (Sigma, F1804), goat anti-mouse IgG antibody (H + L) Alexa Fluor 647 (Abcam, ab150119), goat anti-rabbit IgG antibody (H + L) Alexa Fluor 647 (Abcam, ab150083), goat anti-mouse IgG antibody (H + L) Alexa Fluor 555 (Abcam, ab150118) and goat anti-rabbit IgG antibody (H + L) Alexa Fluor 555 (Abcam, ab150086).

### Quantification and statistical analysis

Microsoft Excel and Prism 6 were used for statistical analysis. Mean ± s.e.m. is shown in all graphs. Unpaired two-tailed Student’s *t*-test was used to determine the significant differences between the two conditions in Figures 2F, 3D, 4B–4E, 4H, 4K–4M, S1A, S5E, S5H, and S5I). p < 0.05 was considered a significant difference. ∗, 0.01 < p < 0.05; ∗∗, 0.001 < p < 0.01; ∗∗∗, p < 0.001. One-way ANOVA calculated adjusted p values to compare more than two conditions in Figures 1E, 1H, 3H, S1D, S1E, and S6K. Tukey test was used for multiple comparisons and the p value was adjusted to account for multiple comparisons. Two-way ANOVA calculated adjusted p values to compare atmosphere and 5% CO_2_ conditions by Sidak test in Figure 1E.

## Data Availability

• All original data underlying this manuscript can be accessed from the Stowers Original Data Repository at: http://www.stowers.org/research/publications/libpb-1281. • All codes used for plugins in Fiji are available at: https://github.com/jouyun. • All software within this manuscript is referenced in the key resources table.

## Supplemental information

Document S1. Figures S1–S7

Table S1. Formulations of media used in the current study, related to Figure 1

Table S2. Screen of electroporation conditions for live cells and X1, related to Figure 3

Table S3. Sequences of indicated genes to synthesize mRNA *in vitro*, related to Figure 4
