# Supplementary material for: Pluripotency retention and exogenous mRNA introduction in planarian stem cells in culture
Source: iScience. 2023 Jan 20;26(2):106001. doi: 10.1016/j.isci.2023.106001 (PMC9971864; doi:10.1016/j.isci.2023.106001)
Supplement: Document S1. Figures S1–S7 [file mmc1.pdf]

iScience, Volume *26*

Supplemental information

Pluripotency retention and exogenous mRNA introduction in planarian stem cells in culture

Kai Lei, Wenya Zhang, Jiajia Chen, Sean A. McKinney, Eric J. Ross, Heng-Chi Lee, and Alejandro Sánchez Alvarado

Supplementary information

Figure S1. Viability of X1(FS) cells cultured in vitro, related to Figure 1. (a) Percentages of live cells (Propidium Iodide-negative) among 23 media, +/- 5% CO_2, after 1 day of culture. Data are represented as mean ± SEM. The student's t-test was used to calculate the p values between the ambient atmosphere and 5% CO_2 for each medium. *, $0.01 < p < 0.05$; **, $0.001 < p < 0.01$; ***, $p < 0.001$. Three replicates were assayed, n=500 to 1200. **(b)** Four representative images showing long cellular processes from cells after 6 days of culture in L15 without 5% CO_2. Scale bar, 20 µm. **(c)** X1(FS) cells were cultured in IPM + 5% CO_2 for 2 days. Representative images of apoptotic cells (Annexin V, green, arrowheads) co-stained with the pan-neoblast marker *smedwi-1* (magenta), n=37. Two independent replicate experiments were performed. No co-labeling was observed, suggesting neoblasts examined in the study were viable. Scale bar, 20 µm. **(d)** Percentage of *smedwi-1*+ neoblasts after 3 days of culture in indicated media + 5% CO_2. Data are represented as mean ± SEM. Adjusted p values were calculated by one-way ANOVA with the Tukey test. *, $0.01 < p < 0.05$; **, $0.001 < p < 0.01$. **(e)** Percentage of *PCNA*+ neoblasts after 1 day of culture in indicated media + 5% CO_2. Data are represented as mean ± SEM. Adjusted p values were calculated by one-way ANOVA with the Tukey test. ***, $p < 0.001$.

Figure S2. Sexual hosts are rescued and reconstituted by transplanting cultured asexual X1(FS) cells, related to Figure 1. (a, b) Determine the number of X1(FS) cells needed for efficient colony expansion. **(a)** Percentage of lethally irradiated hosts

displaying robust neoblast colony expansion following transplantation with the indicated numbers of sorted X1(FS) cells. At 7 days post-transplantation (dpt), > 80% of all hosts displayed colony expansion when 1,000 X1(FS) were transplanted. **(b)** Representative images of hosts transplanted with X1(FS) cells at 7 dpt. *smedwi-1*+ neoblasts: green. DAPI: blue. Scale bar, 200 µm. Ten animals were assayed per condition. **(c)** Representative images showing colonies of *smedwi-1*+ neoblasts at 8 days post-transplantation (dpt) cultured in the indicated conditions + 5% CO_2. The numbers indicate the percentage of hosts receiving X1(FS) cells cultured in indicated media + 5% CO_2 for 1, 2, or 3 days that possessed *smedwi-1*+ colonies at 8 dpt. n = 10. Only X1(FS) cells cultured in dGrace's medium + 5% CO_2 did not efficiently form colonies *in vivo*. Scale bar, 200 µm. **(d)** Sequence showing the HpaI enzyme restriction site, which was used to distinguish between the asexual (donor) and sexual (host) biotypes by RFLP analyses (Wagner et al., 2011). **(e)** RFLP data showing the rescue of lethally irradiated sexual worms transplanted with freshly collected, non-cultured X1(FS) cells. **(f, g)** RFLP data showing the rescue of lethally irradiated sexual worms transplanted with 1- and 2-day cultured X1(FS) cells. Data from two independent experiments showed replicate 1 (panel f); replicate 2 (panel g).

Figure S3. Comparison of SiR-DNA sorted cells, related to Figure 2. (a) A plot showing how SiR-DNA-stained cells are displayed without gates in the flow cytometry analysis using SiR-DNA versus side scatter. **(b)** A plot showing how gates were defined to isolate two SiR-DNA staining cell populations based on DNA content (SiR-DNA 4n and 2n). **(c)** *smedwi-1 in situ* staining for neoblasts in two isolated cell populations based on

DNA content (b). SiR-DNA 4n population contains 56.4%±2.6% *smedwi-1*+ neoblasts (also see Fig. 4f) compared to 26.8%±3.2% in SiR-DNA 2n population, p-value = 0.0017. Scale bar, 20 µm. **(d-g)** Plots showing the cell cycle distribution of SiRNeoblasts (SiR-DNA 4n + CT) (d), cells between SiR-DNA 4n and 2n (e), SiR-DNA 2n (f), and all SiR-DNA+ cells (g). Sorted cells were stained with Hoechst 33342. Hoechst 33342+ (square gate) cells were analyzed for cell cycle distribution.

Figure S4. KnockOut DMEM maintains the expression of *smedwi-1* or *tgs-1* independently on differentiated cells, related to Figure 2. (a) Percentage of *smedwi-1*+ or *tgs-1*+ neoblasts within the first three days of culture in KnockOut DMEM + 5% CO_2. **(b)** Percentage of FITC+ cells, *smedwi-1*+ or *tgs-1*+ neoblasts when SiRNeoblasts were co-cultured with X1(FS) within the first three days of culture in KnockOut DMEM + 5% CO_2. Data are represented as mean ± SEM.

Figure S5. Electroporation can deliver exogenous mRNA into neoblasts, related to Figure 3. (a) Flowchart describing electroporation assay steps to screen for best conditions for cell viability and Dextran-FITC delivery efficiency. **(b)** Plots of X1 viability (upper) and electroporation efficiency (lower) with IPM as the electroporation buffer to deliver Dextran-FITC at 120V compared to 0 V controls. **(c)** Representative images of sorted Dextran-FITC^{low} and Dextran_FITC^{high} cells indicate successful delivery of Dextran-FITC at 120V. Scale bar, 10 µm. **(d)** Viability (blue) and electroporation efficiency (red) on X1 cells after using IPM as electroporation buffer. The viability of X1 is indicated by the percentage of X1 cells within all Hoechst 33342+ cells. **(e)** %*smedwi-1*+ neoblasts

in X1(FS) cells after 100V, 110V, and 120V electroporation immediately (black column) and after 1 day of culture in KnockOut DMEM + 5% CO_2 (white column). Four random fields were assayed per condition. Data are represented as mean ± SEM. The student's t-test was used to calculate the p values. *, $0.01 < p < 0.05$; **, $0.001 < p < 0.01$. N>40. **(f)** Electroporated X1(FS) cells receiving greater than 100 V failed to form colonies following transplantation. Ten animals were assayed per condition. **(g)** ISH for *tdTomato* mRNA in cultured neoblasts at 20 hours post electroporation at 110V after RNase A treatment. 1% (V/V) RNase A was used for 30min incubation. Scale bar, 10 μm. **(h)** The statistical analysis for *tdTomato* mRNA+ cells at 20 hours post electroporation at 110V in the presence or absence of RNase A. Data are represented as mean ± SEM. The student's t-test was used to calculate the p values. **, $0.001 < p < 0.01$; n.s., not significant. **(i)** *ISH* for *NanoLuc* mRNA and statistical analysis for *NanoLuc* mRNA+ cells in cultured neoblasts at 20 hours post electroporation at 110V. Scale bar, 10 μm. Data are represented as mean ± SEM. The student's t-test was used to calculate the p values. **, $0.001 < p < 0.01$.

Figure S6. Detection of NanoLuc in planarian cells cultured *in vitro*, related to Figure 4. (a) ISH for *NanoLuc* mRNA in cultured SiRNeoblasts at one-day post-transfection through *Tran*sIT in the presence or absence of RNase A. SiRNeoblasts not electroporated were used as negative controls. 5% (V/V) RNase A was incubated for 2 hours. Scale bar, 10 μm. **(b)** Comparison of the fluorescent intensity of *NanoLuc* mRNA with or without poly-A tail in cultured SiRNeoblasts at 1 day post-transfection. The top panel shows the ISH signal of *NanoLuc* mRNA with poly A tail. The middle panel shows

the ISH signal of *NanoLuc* mRNA without poly A tail. The bottom panel shows the ISH signal of *NanoLuc* mRNA without poly A tail after brightness adjustment to compare the fluorescent intensity inside cells with those in the medium. Scale bar, 10 μm. **(c-f)** Expression of *NanoLuc* (c), *mCherry_4* (d), *NanoLuc-mCherry* (e), and *histone3.3-2×flag* (f) mRNA in cultured 1000,000 planarian live cells and 293T cells transfected by *Trans*IT at 1 div through Western blot. The antigens against by antibodies are indicated on the left of the blots. The molecular weights are labeled on the right of the blots. **(g-i)** Antibody immunofluorescence staining in 293T cells (g), 20,000 SiRNeoblasts (i), and bulk live cells (h) transfected with *NanoLuc* (c), *mCherry_4* (d), *NanoLuc-mCherry* (e), and *histone3.3-2×flag* (f) mRNA by *Trans*IT at 1 div. Scale bar, 50 μm. **(j)** NanoLuc+ cells are *smedwi-1* low or negative cells. Scale bar, 10 μm. **(k)** Expression of *NanoLuc* mRNA in 200,000 Hoechst 33342 staining cells (X1, X2+Xins, all live cells) and live cells without staining transfected by *Trans*IT in KnockOut DMEM + supplements + 5%CO_2. Data are represented as mean ± SEM. One-way ANOVA calculated adjusted p values to compare the NanoLuc levels in different cell types. Tukey test was used for multiple comparisons and the p value was adjusted to account for multiple comparisons. *, $0.01 < p < 0.05$; n.s., not significant.

Figure S7. NanoLuc positive cells were *smedwi-1* negative and SMEDWI-1 low in cultured SiRNeoblasts, related to Figure 4. All 52 NanoLuc+ cells captured were stained with *smedwi-1* RNA probe (red) or SMEDWI-1 antibody (magenta). CTG was in Green, indicating the cytoplasm membrane's integrity during the fixation. Scale bar, 10 μm.

Figure S1

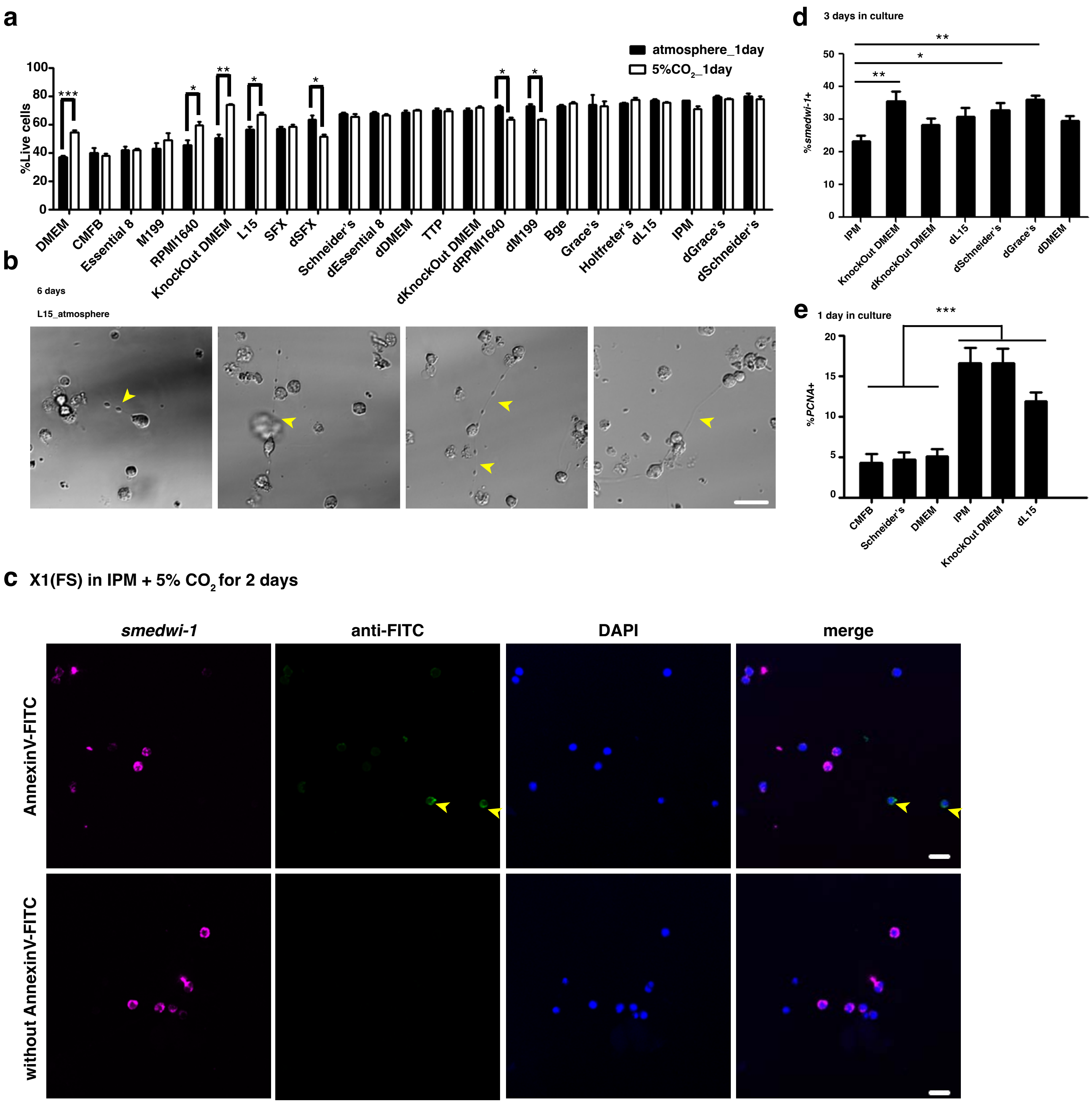
a
atmosphere_1day
5%CO2_1day
%Live cells
100
80
60
40
20
0
***
*
**
*
*
*
*
DMEM
CMFB
Essential 8
M199
RPMI1640
KnockOut DMEM
L15
SFX
dSFX
Schneider's
dEssential 8
dDMEM
TTP
dKnockOut DMEM
dRPMI1640
dM199
Bge
Grace's
Holtfreter's
dL15
IPM
dGrace's
dSchneider's
b
6 days
L15_atmosphere
c X1(FS) in IPM + 5% CO2 for 2 days
smedwi-1
anti-FITC
DAPI
merge
AnnexinV-FITC
without AnnexinV-FITC
d
3 days in culture
%smedwi-1+
50
40
30
20
10
0
**
*
**
IPM
KnockOut DMEM
dKnockOut DMEM
dL15
dSchneider's
dGrace's
dDMEM
e
1 day in culture
***
%PCNA+
20
15
10
5
0
CMFB
Schneider's
DMEM
IPM
KnockOut DMEM
dL15

Figure S2

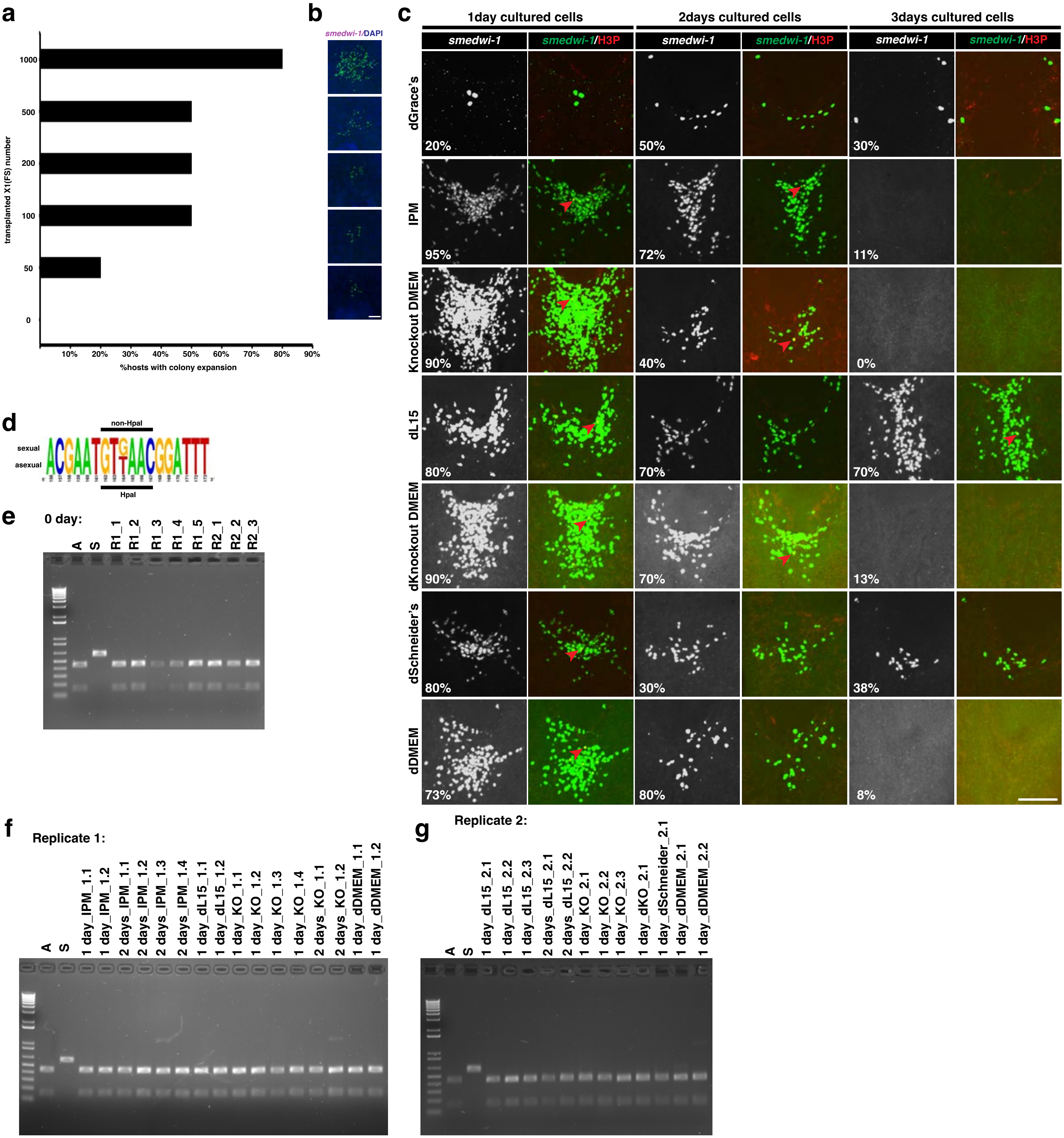

a
transplanted X1(FS) number
1000
500
200
100
50
0
10%
20%
30%
40%
50%
60%
70%
80%
90%
%hosts with colony expansion
b
smedwi-1/DAPI
c
1day cultured cells
2days cultured cells
3days cultured cells
smedwi-1
smedwi-1/H3P
dGrace's
IPM
Knockout DMEM
dL15
dKnockout DMEM
dSchneider's
dDMEM
20%
50%
30%
95%
72%
11%
90%
40%
0%
80%
70%
70%
90%
70%
13%
80%
30%
38%
73%
80%
8%
d
non-HpaI
sexual
asexual
HpaI
e
0 day:
A
S
R1_1
R1_2
R1_3
R1_4
R1_5
R2_1
R2_2
R2_3
f
Replicate 1:
A
S
1 day_IPM_1.1
1 day_IPM_1.2
2 days_IPM_1.1
2 days_IPM_1.2
2 days_IPM_1.3
2 days_IPM_1.4
1 day_dL15_1.1
1 day_dL15_1.2
1 day_KO_1.1
1 day_KO_1.2
1 day_KO_1.3
1 day_KO_1.4
2 days_KO_1.1
2 days_KO_1.2
1 day_dDMEM_1.1
1 day_dDMEM_1.2
g
Replicate 2:
A
S
1 day_dL15_2.1
1 day_dL15_2.2
1 day_dL15_2.3
2 days_dL15_2.1
2 days_dL15_2.2
1 day_KO_2.1
1 day_KO_2.2
1 day_KO_2.3
1 day_dKO_2.1
1 day_dSchneider_2.1
1 day_dDMEM_2.1
1 day_dDMEM_2.2

Figure S3

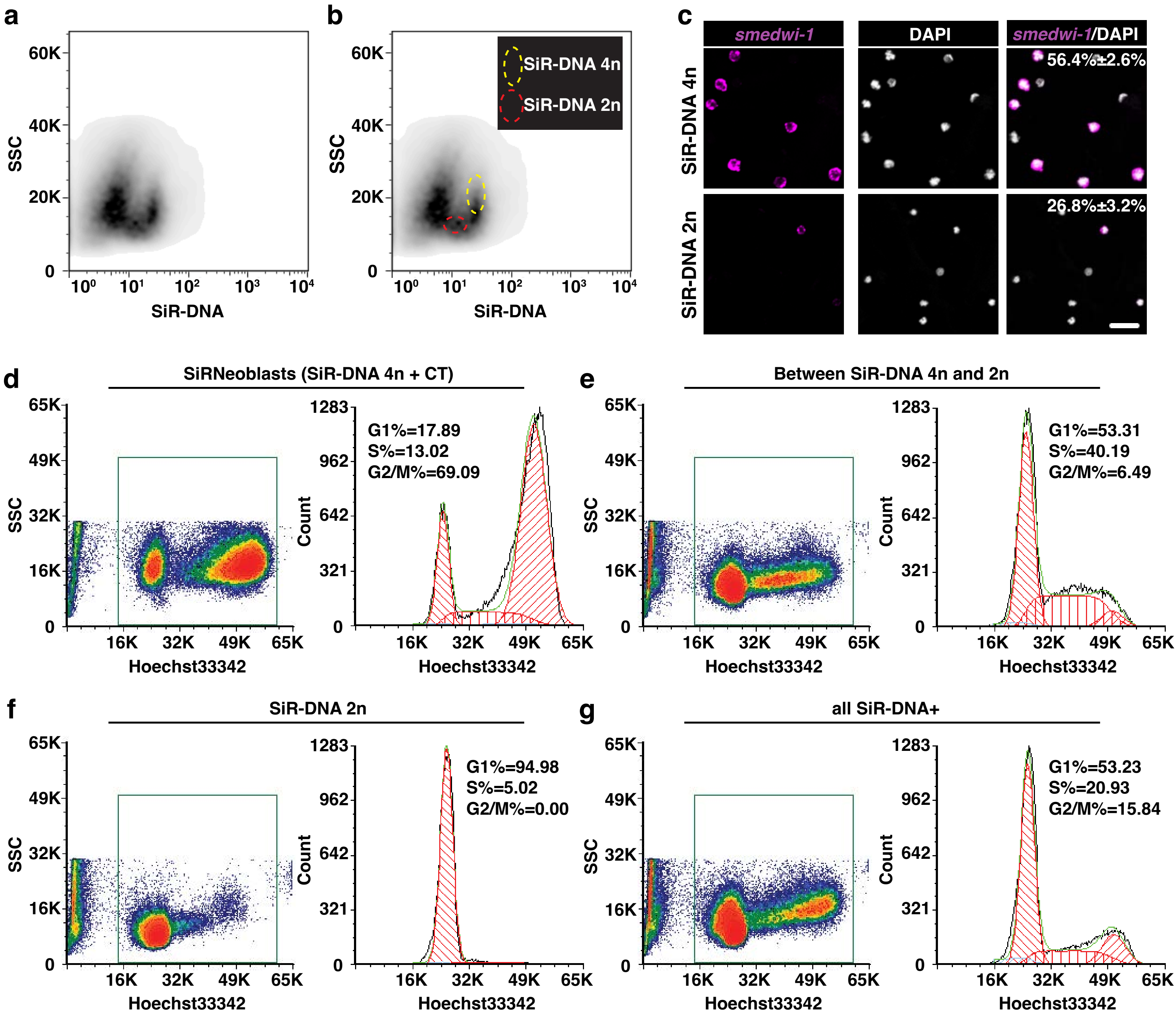

a
60K
40K
20K
0
SSC
10⁰ 10¹ 10² 10³ 10⁴
SiR-DNA
b
SiR-DNA 4n
SiR-DNA 2n
SSC
SiR-DNA
c
smedwi-1
DAPI
smedwi-1/DAPI
SiR-DNA 4n
56.4%±2.6%
SiR-DNA 2n
26.8%±3.2%
d
SiRNeoblasts (SiR-DNA 4n + CT)
65K
49K
32K
16K
SSC
16K 32K 49K 65K
Hoechst33342
1283
962
642
321
Count
G1%=17.89
S%=13.02
G2/M%=69.09
e
Between SiR-DNA 4n and 2n
G1%=53.31
S%=40.19
G2/M%=6.49
f
SiR-DNA 2n
G1%=94.98
S%=5.02
G2/M%=0.00
g
all SiR-DNA+
G1%=53.23
S%=20.93
G2/M%=15.84

Figure S4

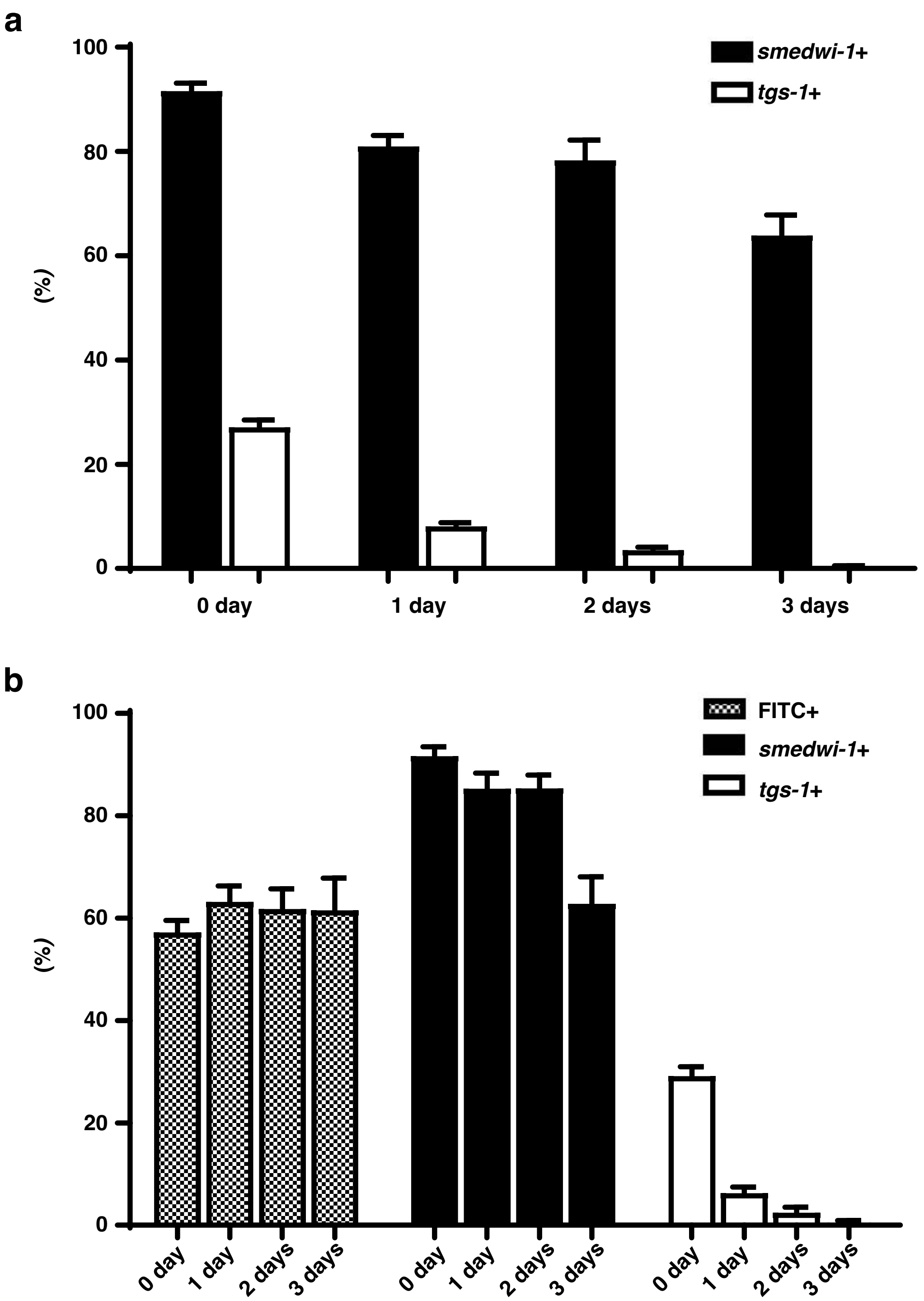
a
100
80
60
40
20
0
(%)
smedwi-1+
tgs-1+
0 day
1 day
2 days
3 days
b
100
80
60
40
20
0
(%)
FITC+
smedwi-1+
tgs-1+
0 day
1 day
2 days
3 days
0 day
1 day
2 days
3 days
0 day
1 day
2 days
3 days

Figure S5

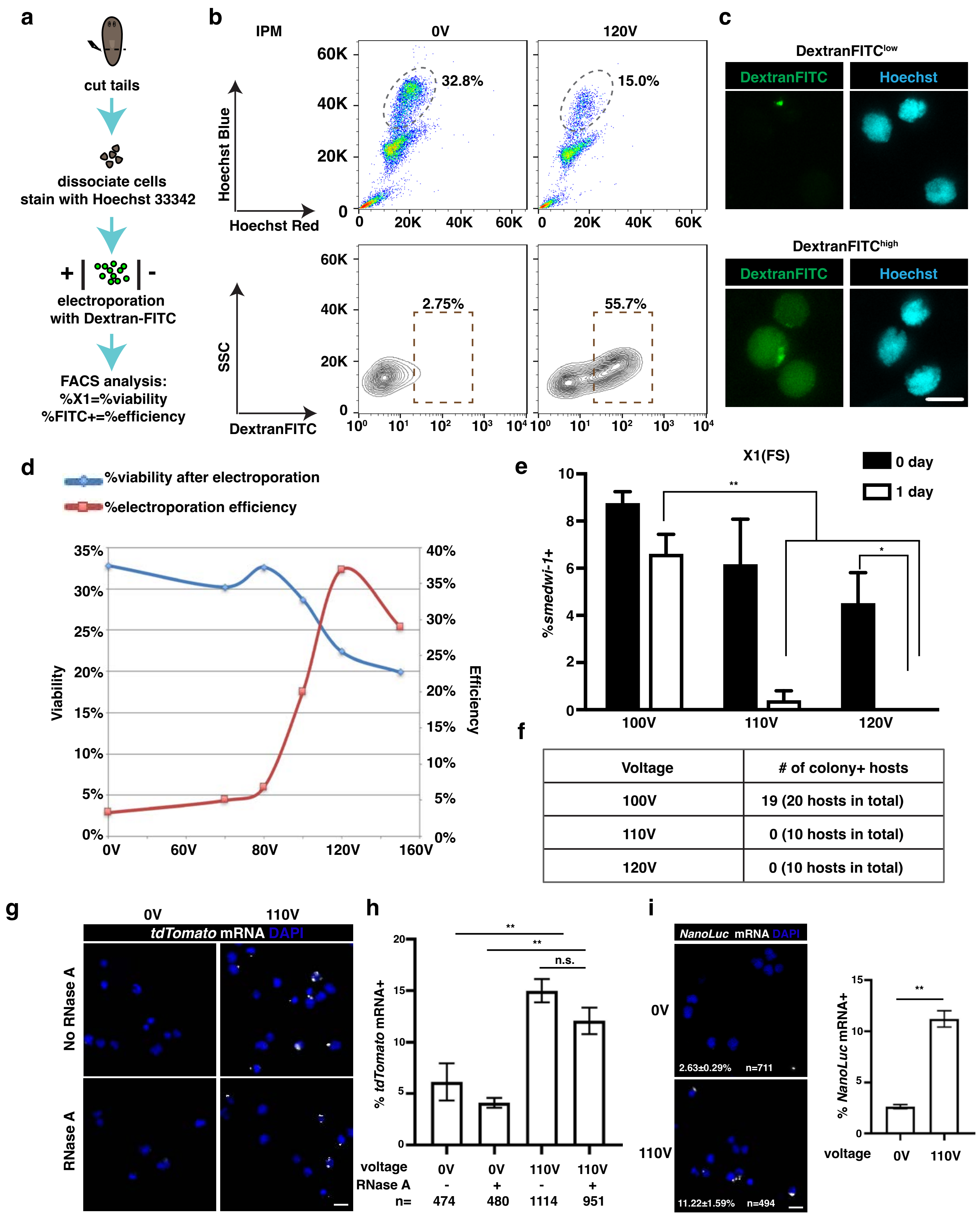

a
cut tails
dissociate cells
stain with Hoechst 33342
electroporation
with Dextran-FITC
FACS analysis:
%X1=%viability
%FITC+=%efficiency
b
IPM
0V
120V
Hoechst Blue
Hoechst Red
32.8%
15.0%
2.75%
55.7%
SSC
DextranFITC
c
DextranFITC^low
DextranFITC^high
DextranFITC
Hoechst
d
%viability after electroporation
%electroporation efficiency
Viability
Efficiency
e
X1(FS)
0 day
1 day
%smedwi-1+
100V
110V
120V
f
Voltage | # of colony+ hosts
100V | 19 (20 hosts in total)
110V | 0 (10 hosts in total)
120V | 0 (10 hosts in total)
g
0V
110V
tdTomato mRNA DAPI
No RNase A
RNase A
h
% tdTomato mRNA+
voltage
RNase A
n=
474
480
1114
951
n.s.
i
NanoLuc mRNA DAPI
0V
110V
2.63±0.29% n=711
11.22±1.59% n=494
% NanoLuc mRNA+
voltage

Figure S6

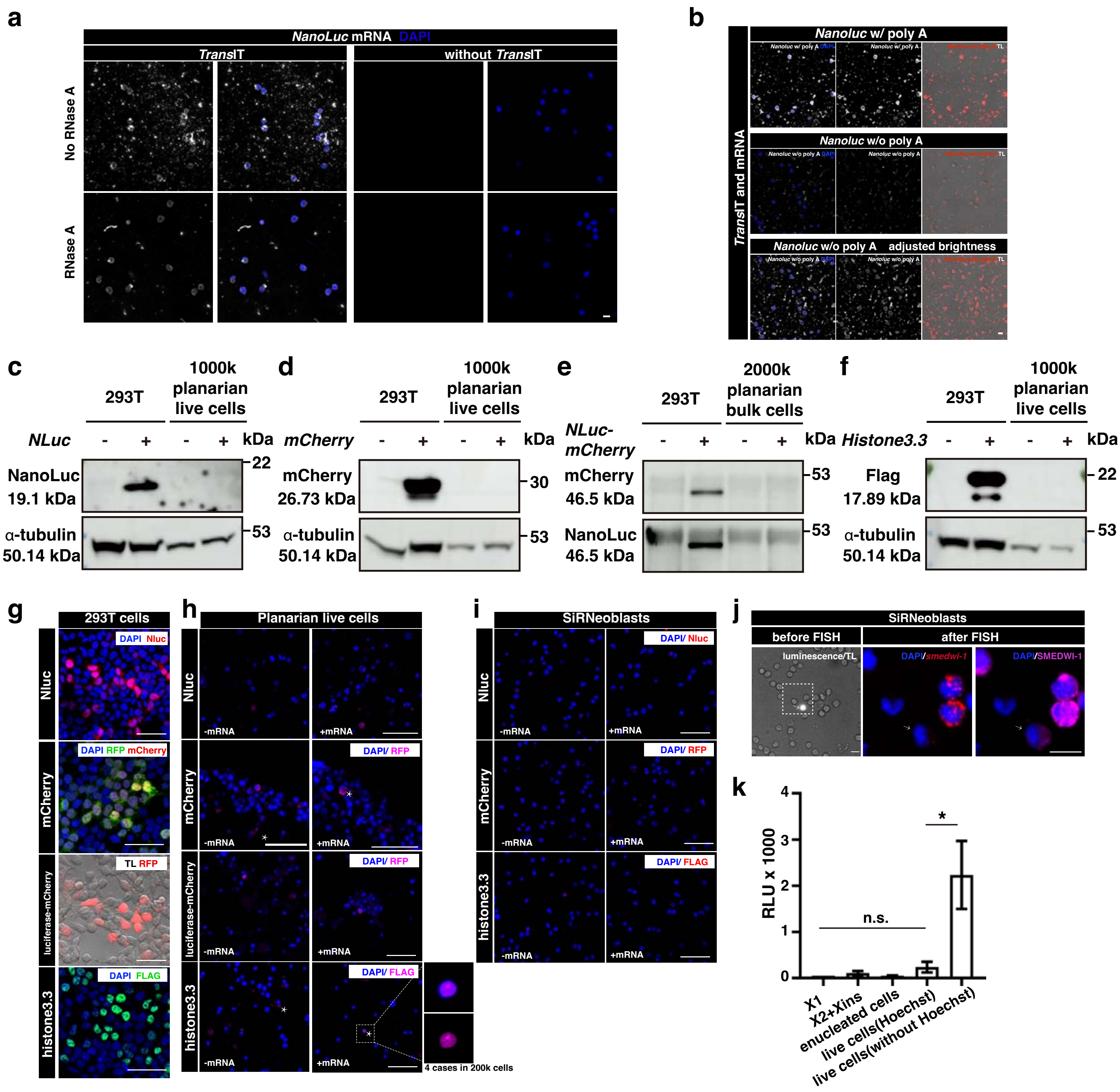
a
NanoLuc mRNA DAPI
TransIT
without TransIT
No RNase A
RNase A
b
TransIT and mRNA
Nanoluc w/ poly A
Nanoluc w/o poly A
Nanoluc w/o poly A adjusted brightness
c
293T
1000k planarian live cells
NLuc
- + - +
kDa
NanoLuc 19.1 kDa
α-tubulin 50.14 kDa
22
53
d
293T
1000k planarian live cells
mCherry
- + - +
kDa
mCherry 26.73 kDa
α-tubulin 50.14 kDa
30
53
e
293T
2000k planarian bulk cells
NLuc-mCherry
- + - +
kDa
mCherry 46.5 kDa
NanoLuc 46.5 kDa
53
53
f
293T
1000k planarian live cells
Histone3.3
- + - +
kDa
Flag 17.89 kDa
α-tubulin 50.14 kDa
22
53
g
293T cells
Nluc
mCherry
luciferase-mCherry
histone3.3
DAPI Nluc
DAPI RFP mCherry
TL RFP
DAPI FLAG
h
Planarian live cells
Nluc
mCherry
luciferase-mCherry
histone3.3
-mRNA
+mRNA
DAPI/ RFP
DAPI/ FLAG
4 cases in 200k cells
i
SiRNeoblasts
Nluc
mCherry
histone3.3
-mRNA
+mRNA
DAPI/ Nluc
DAPI/ RFP
DAPI/ FLAG
j
SiRNeoblasts
before FISH
after FISH
luminescence/TL
DAPI/smedwi-1
DAPI/SMEDWI-1
k
RLU x 1000
0
1
2
3
4
n.s.
*
X1
X2+Xins
enucleated cells
live cells(Hoechst)
live cells(without Hoechst)

Figure S7

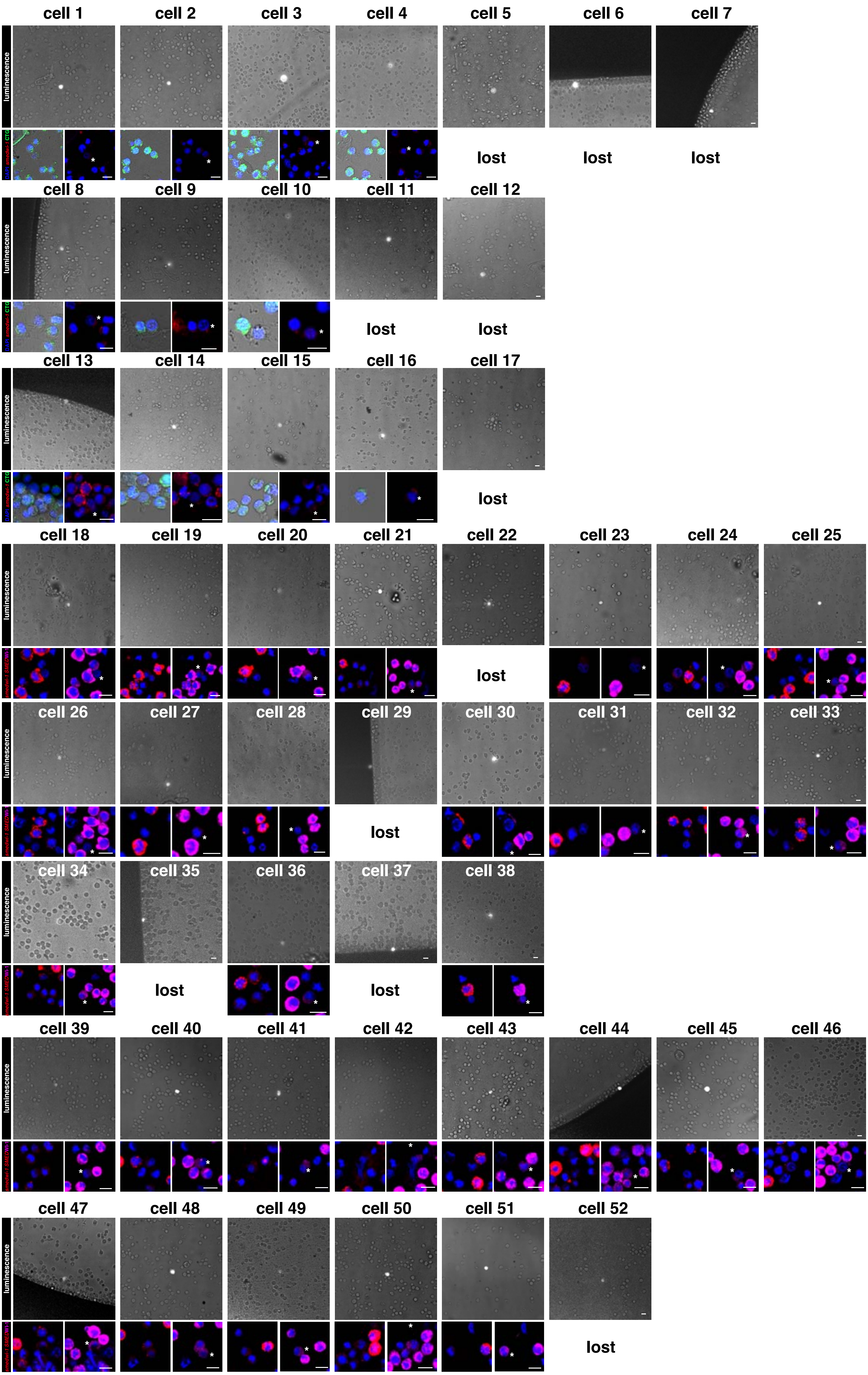
cell 1
cell 2
cell 3
cell 4
cell 5
cell 6
cell 7
luminescence
lost
lost
lost
cell 8
cell 9
cell 10
cell 11
cell 12
lost
lost
cell 13
cell 14
cell 15
cell 16
cell 17
lost
cell 18
cell 19
cell 20
cell 21
cell 22
cell 23
cell 24
cell 25
lost
cell 26
cell 27
cell 28
cell 29
cell 30
cell 31
cell 32
cell 33
lost
cell 34
cell 35
cell 36
cell 37
cell 38
lost
lost
cell 39
cell 40
cell 41
cell 42
cell 43
cell 44
cell 45
cell 46
cell 47
cell 48
cell 49
cell 50
cell 51
cell 52
lost
